# Neuroprotection by ADAM10 inhibition requires TrkB signaling in the Huntington’s disease hippocampus

**DOI:** 10.1007/s00018-024-05382-1

**Published:** 2024-08-07

**Authors:** Andrea Scolz, Elena Vezzoli, Michela Villa, Francesca Talpo, Jessica Cazzola, Francesca Raffin, Chiara Cordiglieri, Andrea Falqui, Giuseppe Pepe, Vittorio Maglione, Dario Besusso, Gerardo Biella, Chiara Zuccato

**Affiliations:** 1 Department of Biosciences, University of Milan, Milan, Italy; 2 Istituto Nazionale di Genetica Molecolare “Romeo ed Enrica Invernizzi”, Milan, Italy; 3 Department of Biology and Biotechnology “Lazzaro Spallanzani”, University of Pavia, Pavia, Italy; 4 Interdisciplinary Centre for Nanostructured Materials and Interfaces (CIMaINa), Department of Physics, University of Milan, Milan, Italy; 5 IRCCS Neuromed, Pozzilli, Isernia Italy; 6 Present Address: Advanced Light and Electron Microscopy BioImaging Centre (ALEMBIC), IRCCS San Raffaele Scientific Institute, Milan, Italy

**Keywords:** Huntingtin, Synaptic dysfunctions, ADAM10, BDNF

## Abstract

Synaptic dysfunction is an early pathogenic event leading to cognitive decline in Huntington’s disease (HD). We previously reported that the active ADAM10 level is increased in the HD cortex and striatum, causing excessive proteolysis of the synaptic cell adhesion protein N-Cadherin. Conversely, ADAM10 inhibition is neuroprotective and prevents cognitive decline in HD mice. Although the breakdown of cortico-striatal connection has been historically linked to cognitive deterioration in HD, dendritic spine loss and long-term potentiation (LTP) defects identified in the HD hippocampus are also thought to contribute to the cognitive symptoms of the disease. The aim of this study is to investigate the contribution of ADAM10 to spine pathology and LTP defects of the HD hippocampus. We provide evidence that active ADAM10 is increased in the hippocampus of two mouse models of HD, leading to extensive proteolysis of N-Cadherin, which has a widely recognized role in spine morphology and synaptic plasticity. Importantly, the conditional heterozygous deletion of ADAM10 in the forebrain of HD mice resulted in the recovery of spine loss and ultrastructural synaptic defects in CA1 pyramidal neurons. Meanwhile, normalization of the active ADAM10 level increased the pool of synaptic BDNF protein and activated ERK neuroprotective signaling in the HD hippocampus. We also show that the ADAM10 inhibitor GI254023X restored LTP defects and increased the density of mushroom spines enriched with GluA1-AMPA receptors in HD hippocampal neurons. Notably, we report that administration of the TrkB antagonist ANA12 to HD hippocampal neurons reduced the beneficial effect of GI254023X, indicating that the BDNF receptor TrkB contributes to mediate the neuroprotective activity exerted by ADAM10 inhibition in HD. Collectively, these findings indicate that ADAM10 inhibition coupled with TrkB signaling represents an efficacious strategy to prevent hippocampal synaptic plasticity defects and cognitive dysfunction in HD.

**Supplementary Information:**

The online version contains supplementary material available at 10.1007/s00018-024-05382-1.

## Introduction

Huntington’s disease (HD) is a dominantly inherited disorder of the central nervous system that typically becomes evident in mid-life [1]. Carriers of a cytosine-adenine-guanine (CAG) trinucleotide expansion of at least 36 repeats in the exon 1 of the huntingtin (*HTT*) gene experience synaptic dysfunction and cognitive deficiencies from the prodromal stage of the disease, long before the emergence of motor symptoms [2, 3]. The breakdown of cortico-striatal connection has been historically linked to cognitive decline in HD [4, 5]. Moreover, evidence in mouse models supports the role of hippocampal-mediated cognitive abnormalities in HD. In fact, reduced spine density and impairment in N-methyl-D-aspartate receptor (NMDAR)-dependent long-term potentiation (LTP) at the Schaffer's collateral-CA1 synapse have both been associated to cognitive decline in HD mice [6–11]. Notably, clinical studies in HD patients argue in favor of hippocampal involvement in the cognitive deficit, consistent with what has been reported in the mouse models of HD [12, 13].

Spine loss and LTP impairments in the HD hippocampus have been predominantly associated with a reduction in the tropomyosin receptor kinase B (TrkB) signaling pathway due to diminished transcription and axonal transport of Brain-Derived Neurotrophic Factor (BDNF) [10, 14]. In HD, decreased BDNF/TrkB signaling has also been identified as the cause of reduced α-amino-3-hydroxy-5-methyl-4-isoxazolepropionic acid (AMPA) receptor lateral surface diffusion, a crucial mechanism in LTP [10]. The observation that supplying BDNF restores LTP defects in HD hippocampal brain slices confirms the role of BDNF/TrkB pathway in hippocampal plasticity and in HD [15].

Beyond BDNF, the hippocampus is enriched with A Disintegrin and Metalloproteinase Domain-Containing Protein 10 (ADAM10), a recently discovered binding partner of HTT at the synapse with a consolidated role in spine formation, stabilization, and synaptic plasticity [16–19]. Initially produced as a 95 kDa zymogen, ADAM10 matures into a 60 kDa active protease (m-ADAM10) that regulates synaptic cell adhesion by exerting a proteolytic effect on the ectodomain of a plethora of neuronal substrates, including amyloid precursor protein (APP), the cellular prion protein, neuroligin 1, neural cell adhesion molecule (NCAM), nectin 1, ephrin-A2 and -A5, and N-Cadherin (N-CAD) [17, 20]. The recent discovery that ADAM10 binds to presynaptic proteins implicated in the transport, positioning, and release of synaptic vesicles (SVs) consolidates its role in the plasticity of synaptic transmission [19].

Considering the crucial role of this protease in the brain, dysfunction in ADAM10 activity has been linked to the development of brain disorders, including Alzheimer’s disease, Fragile X syndrome, prion disease and, ultimately, HD [17–19]. Indeed, we reported that wild-type HTT binds to active ADAM10 [16, 18]. Conversely, reduced affinity of mutant HTT to the active enzyme causes its accumulation at the synapse, resulting in excessive N-CAD proteolysis in the HD cortex and striatum, leading to synapse loss and cognitive decline in HD mice [18, 19]. Protection from synaptic structural and functional impairments and amelioration of cognitive defects were instead observed when the level of active ADAM10 was normalized in the HD mouse brain through genetic, molecular, and chemical approaches [18, 19].

Due to the ever-increasing burden that cognitive dysfunctions have taken on in HD patients and the lack of effective drug treatments, the aim of this study is to investigate the impact of ADAM10 inhibition strategies on morphological, molecular, and functional defects of the HD hippocampus. The experiments outlined below provide evidence of the detrimental role played by hyperactive ADAM10 in the HD hippocampal synapse. Furthermore, we demonstrate that inhibition of ADAM10 prevents structural and functional synaptic defects in the HD hippocampus. We also show that neuroprotection by ADAM10 inhibition requires a functional BDNF/TrkB pathway in the HD synapse and supports ADAM10 inhibition coupled with TrkB signaling as a strategy to prevent cognitive symptoms in HD.

## Methods

### HD mouse models

Wild-type and R6/2 mice on a B6CBAF1/J background and zQ175DN mice on a C57BL6/J background were purchased from the Jackson Laboratory. Both males and females were included in the study. Genotyping of R6/2 (B6CBAF1/J) and zQ175DN (C57BL/6 J) mouse colonies (~ 150 CAG and 175 repeats, respectively) was performed by PCR of genomic DNA obtained from tail samples (Nucleo Spin Tissue, Macherey–Nagel, Cat. No. 740952.250) at weaning and following sacrifice for verification. CAG repeats of R6/2 and zQ175DN mice were sized as described in [18].

### Generation of R6/2-A10cKO mice

R6/2 mice (B6CBAF1/J) were generated by crossing R6/2 males with wild-type females (B6CBAF1/J). The A10cKO colony was maintained by crossing heterozygous ADAM10 floxed mice (*Adam10*^*Flox/+*^) (C57BL/6 J × 129 S6) with CaMKIIalpha-Cre recombinase transgenic mice (C57BL/6 J × 129 S6). A10cKO mice exhibited a normal phenotype and normal fertility. From the crossing between heterozygous A10cKO and R6/2 mice, we tested 4 genotypes of the F1, including WT, R6/2, R6/2-A10cKO, and A10cKO, which are on the same mixed genetic background. Mice were genotyped as previously described in [18].

### Treatment of mice with TAT peptides

Wild-type and R6/2 mice at 12 weeks of age received 2 i.p. injections 24 h apart of TAT-Pro-ADAM10^709–729^ (2 nmol/g) or TAT-Ala-ADAM10^709–729^ peptide (2 nmol/g) as described in [18]. Animals were euthanized 24 h after the second injection by cervical dislocation, and the brains were rapidly removed for dissection of the hippocampal tissues.

### Primary mouse hippocampal neurons

The hippocampus was isolated from E18 mouse fetuses in ice-cold Hybernate™-E medium (Gibco™, Thermo Fisher Scientific, Cat. No. A1247601). The tissue was incubated for 15 min at 37 °C in pre-warmed dissociation buffer (PBS1X, papain 500 µg/mL, DNase I 2 U/mL, 5 mM glucose). Papain was then inactivated by adding PBS1X supplemented with 10% fetal bovine serum (FBS) (Euroclone,  Cat. No. EUS028597).﻿ After centrifugation for 3 min at 800 rpm, the solution was removed, and tissues were then mechanically dissociated in Neurobasal medium (Gibco™, Thermo Fisher Scientific, Cat. No. 21103049) supplemented with L-glutamine (GlutaMax from Gibco™, Cat. No. 35050061), N-2 supplement (Gibco™, ﻿﻿﻿﻿﻿﻿﻿﻿﻿T﻿hermo ﻿﻿Fisher﻿ ﻿Scientific,﻿ ﻿Cat. No. 17502048),﻿ ﻿B-27 supplement (Gibco™, Thermo Fisher Scientific, Cat. No. 17504044) and 10% FBS. Neurons were plated on poly-D-lysine-coated (Sigma-Aldrich, Cat. No. p6407-5MG) 12-mm glass coverslips (VWR, Cat. No. 631–1577) at a density of 1 × 10^5^ cells/cm^2^. After 12–24 h from plating, FBS withdrawal was performed, and neurons were maintained in supplemented Neurobasal medium without FBS for up to 14 days in vitro (DIV14). Partial medium replacement was performed every 48 h. Treatment with the ADAM10 inhibitor GI254023X 1 µM (CliniSciences Cat. No.  HY-19956-5mg) was carried out from DIV6 until DIV14 during medium replacement. The TrkB blocker ANA12 (10 µM) (Tocris, Bio-Techne, Cat. No. 4781) was co-administered during medium replacement for 48 h before cell fixation at DIV14.

### Preparation of total protein lysates and synaptosomes

Total protein lysates were prepared in RIPA buffer (50 mM Tris–HCl pH 8, 150 mM NaCl, 0.1% sodium dodecyl sulfate (SDS), 1% Nonidet P40, 0.5% sodium deoxycholate) with 1 mM phenylmethylsulfonyl fluoride (PMSF) and 1 × protease inhibitor cocktail (Thermo Fisher Scientific, Cat. No. 1861281). Lysates were cleared by centrifugation for 30 min at 12,000 g and 4 °C. Synaptosomes were prepared by using Syn-PER Reagent (Thermo Fisher Scientific, Cat. No. 87793). Protein concentration was determined with the BCA assay (Thermo Fisher Scientific, Cat. No. 23225).

### SDS-PAGE and Western Blot

20–60 μg of proteins were loaded on a 10% SDS-PAGE gel. Separated proteins were transferred onto a nitrocellulose membrane (Bio-Rad, Cat. No. 1704158) by means of the Trans-blot Turbo Transfer System (Bio-Rad) (High Molecular Weight protocol: 2.5 A constant; up to 25 V; 10 min), blocked with 5% nonfat milk (Bio-Rad, Cat. No. 1706404) in TBS1X and 0.1% Tween 20 (TBST) and incubated with rabbit polyclonal anti-ADAM10 antibody EPR5622 (1:1000 in TBST; Abcam, Cat. No. ab124695), mouse monoclonal anti-N-CAD antibody (1:1000 in TBST; Becton Dickinson Transduction Laboratories, Cat. No.610921), rabbit polyclonal anti-total-ERK1/2 antibody (1:2000 in TBST; Cell Signaling, Cat. No. 9102), rabbit polyclonal anti-phospho-ERK1/2 antibody (1:2000 in TBST; Cell Signaling, Cat. No. 9101), mouse monoclonal anti-βIII-Tubulin antibody (1:1000 in TBST; Promega, Cat. No. G7121), and mouse monoclonal anti-α-Tubulin antibody (1:5000 in TBST; Millipore, Cat. No. T9026) at 4 °C overnight. After washing, filters were incubated for 1 h at room temperature (RT) with a peroxidase conjugate secondary antibody (1:3000 in 5% nonfat milk; goat anti-rabbit HRP, Bio-Rad Cat. No. 1706515; goat anti-mouse HRP, Bio-Rad Cat. No. 1706516) and then washed 3 times with TBST. The Clarity Western ECL Substrate (Bio-Rad, Cat. No. 1705061) was used to visualize immunoreactive bands. Blot visualization was performed using the ChemiDoc MP Imaging System from Bio-Rad. Densitometric analyses were performed using Image Lab version 6.0.1.

### Enzyme-linked immunosorbent assay (ELISA)

Due to a high degree of amino acid sequence homology between mouse and human BDNF, the Human BDNF ELISA Kit (Millipore, Cat. No. RAB0026) was used according to the manufacturer’s instructions. Before proceeding with the ELISA assay, synaptosomes underwent an acidification step, which is needed to allow BDNF release from vesicles. 50 µL of synaptosomes were diluted in 150 µL of PBS1X and then incubated with 4 μL of HCl 1N for 15 min at RT. 4 μL of NaOH 1N were then added to each sample to increase the pH of the solution from 4 to 7. All samples were assayed in duplicate, and the average value of each sample was normalized to the total protein concentration.

### RNA preparation and retrotranscription

Total RNA was isolated from 50 to 100 mg of mouse brain tissue using TRIzol reagent according to the manufacturer’s instructions (Invitrogen, Thermo Fisher Scientific, Cat. No. 15596026). RNA concentration was evaluated using the NanoDrop® spectrophotometer, and its integrity was assessed by agarose gel electrophoresis. The *DNA*-free™ DNase Treatment and Removal Reagents (Invitrogen, Thermo Fisher Scientific, Cat. No. AM1906) were used to remove contaminating DNA from RNA preparations, and 500 ng of total RNA was reverse transcribed to single-stranded cDNA by using iScript™ cDNA Synthesis Kit (Bio-Rad, Cat. No. 1708891).

### Real-time qPCR

Reactions were performed in a total volume of 15 μL containing 50 ng cDNA and SsoFast™ EVAGreen Supermix (Bio-Rad, Cat. No 1725204) following the manufacturer’s instructions. Quantitative RT-PCR was performed using a CFX96TM Real-Time System (Bio-Rad). The amplification consisted of the following steps: 95 °C for 3 min, 45 cycles of 30 s at 95 °C, 30 s at 60 °C, and 45 s at 72 °C. Fluorescence was quantified during the annealing step, and product formation was confirmed by melting curve analysis (55–94 °C). Data were analyzed with the CFX Manager software (Bio-Rad).

The following primer pairs (5’-3) were used:

Mouse Actin FW: AGTGTGACGTTGACATCCGTA

Mouse Actin RV: GCCAGAGCAGTAATCTCCTTCT

Mouse ADAM10 FW: GGAAGCTTTAGTCATGGGTCTG

Mouse ADAM10 RV: CTCCTTCCTCTACTCCAGTCAT

Mouse total BDNF FW: TCGTTCCTTTCGAGTTAGCC

Mouse total BDNF RV: TTGGTAAACGGCACAAAAC

Mouse BDNF Ex1 FW: ATCCACTGAGCAAAGCCGAAC

Mouse BDNF Ex2 FW: GTGGTGTAAGCCGCAAAGAAG

Mouse BDNF Ex3 FW: TCTGGCTTGGAGGGCTCCTG

Mouse BDNF Ex4 FW: CAGGAGTACATATCGGCCACCA

Mouse BDNF Ex5 FW: ACCATAACCCCGCACACTCTG

Mouse BDNF Ex6 FW: GGACCAGAAGCGTGACAACA

Mouse BDNF Ex7 FW: CTCTGTCCATCCAGCGCACC

Mouse BDNF Ex8 FW: GGTATGACTGTGCATCCCAGG

Mouse BDNF Ex9a FW: GCTTCCTTCCCACAGTTCCA

Mouse BDNF coding RV: CGCCTTCATGCAACCGAAGT (used for the amplification of BDNF mRNA isoforms).

### Golgi staining

Wild-type, R6/2, R6/2-A10cKO and A10cKO mice at 13 weeks of age were anesthetized with 10 mg/mL 2,2,2-Tribromoethanol (Sigma-Aldrich, Cat. No. T48402) and transcardially perfused using 10/15 mL of saline solution (0.9% NaCl). After perfusion, dissected brains were quickly immersed in Golgi-Cox solution (potassium dichromate 1%, mercuric chloride 1%, and potassium chromate 0.8%) and processed as described in [21]. Stained neurons from the CA1 region of the hippocampus were acquired using a NanoZoomer S60 Digital slide scanner (Hamamatsu C13210-01). Stacks were collected every 0.5 μm with a 40 × objective and n = 3 mice/genotype were analyzed for a total of n = 30 neurons/genotype. The spine density of the proximal apical dendrite area was analyzed (minimum 100 μm from the soma). The second- or third-order dendrite (protruding from its parent apical dendrite) was chosen for spine density quantification. Z-stacks were made from each dendrite in the whole of the analyzed segment. The widths and lengths of dendritic spines were manually measured and categorized into thin spines (with length < 1 µm), stubby spines (with length to width ratios less than or equal to 1 µm), and mushroom spines (with widths > 0.6 μm) according to [22]. To determine the spine density, the software NDP View 2 (Hamamatsu) was used.

### Transmission electron microscopy (TEM)

Sample preparation for TEM imaging was performed as described in [18]. Wild-type, R6/2, R6/2-A10cKO and A10cKO mice at 13 weeks of age (n = 3 mice/genotype) were anesthetized by intraperitoneal injection of 10 mg/mL  2,2,2-Tribromoethanol and transcardially perfused using 2.5% glutaraldehyde (Electron Microscopy Sciences, Cat. No. 16220), and 2% paraformaldehyde (Electron Microscopy Sciences, Cat. No. 19200) as fixatives, both in sodium cacodylate buffer 0.15 M (pH 7.4) (Electron Microscopy Sciences, Cat. No. 12300) and processed as described in [18]. For TEM imaging, ultrathin sections with a thickness of 70 nm were prepared using an UltraCut E Ultramicrotome (Reichert). These sections were then placed on TEM copper grids and imaged by a Tecnai G2 Spirit transmission electron microscope (FEI, Eindhoven, the Netherlands). The microscope operated at an acceleration voltage of 120 kV and was equipped with a lanthanum hexaboride thermionic source, a twin objective lens, and a bottom-mount 11MP Gatan Orius SC1000 CCD camera (Gatan, Pleasanton, USA). Quantitative measurements were performed by ImageJ, version 1.47 (NIH). The selection of the synapse and the analyses were performed by a blinded independent investigator in a genotype-blinded manner. We analyzed n = 3 mice/genotype and n = 60 synapses/genotype. The following parameters were measured: number SVs per μm^2^ presynaptic area, number of docked/reserve/resting vesicles per μm^2^ presynaptic area, and PSD length (nm). For SV distribution, the single vesicle has been manually annotated, and the distance to the active zone was expressed in nm. Docked vesicles were defined as vesicles within 50 nm of the active zone, reserve vesicles as vesicles between 50 and 300 nm from the active zone, and resting vesicles as vesicles beyond 300 nm of the active zone.

### Immunocytochemistry for excitatory synapses analysis

Neurons were fixed in 4% paraformaldehyde for 15 min at RT. Cell membrane permeabilization and blocking of non-specific binding sites were performed in blocking buffer (PBS1X, 0.5% Triton X-100, 5% normal goat serum) for 1 h at RT. Primary antibodies were prepared in diluted blocking buffer (PBS1X, 0.25% Triton X-100, 2.5% normal goat serum) and incubated overnight at 4 °C. Neurons were rinsed three times in PBS1X for 10 min and incubated with secondary antibodies conjugated to Alexa fluorophores for 1 h at RT. Neurons were washed three times in PBS1X and then Hoechst 33258 (Molecular Probes, Invitrogen, Cat. No. H3569) was added for 10 min at RT. Neurons were washed three times in PBS1X and coverslips were mounted with Vectashield Vibrance Antifade Mounting Medium (Vector Labs, Cat. No. H-1700). Images were acquired with a confocal microscope Leica SP5 (LSCM, Leica Microsystems) with a 63 × objective (NA 1.40) or with IN Cell Analyzer 6000 with a 20 × or 40 × objective (GE Healthcare Life Sciences). The following primary antibodies were used: chicken polyclonal anti-Map2 antibody (Abcam, Cat. No. ab5392, 1:2000), mouse monoclonal anti-Bassoon antibody (Enzo Life Science, Cat. No. ADI-VAM-PS003-F, 1:500), rabbit polyclonal anti-Homer1 antibody (GeneTex, Cat. No. GTX103278, 1:500), rabbit polyclonal anti-GluA1 antibody (Millipore, Cat No. AB1504, 1:500). The following secondary antibodies were used: Alexa Fluor 647 goat anti-chicken IgY (Invitrogen, Cat. No. A32933, 1:500) Alexa Fluor 568 goat anti-mouse IgG (Invitrogen, Cat. No. A11004, 1:500), Alexa Fluor 647 goat anti-rabbit IgG (Invitrogen, Cat. No A27040, 1:500). Secondary antibodies were prepared in diluted blocking buffer. Dendrites were identified by Map2 staining. Since primary neuronal cultures can show a heterogeneous distribution, 3–7 fields were captured per well for each biological replicate. Acquired confocal field of images (FoV) were processed with the NIS-Elements software (V.5.30; Lim, Nikon INstruments). Twenty iterations of Richardson-lucy (a specific algorithm for point-scanning-confocal microscopy) deconvolution were applied. The number of synapses in each FoV was measured as Bassoon + /Homer1 + colocalizing puncta and was normalized on the total dendritic length. Since n = 3 biological replicates were performed, a total of 9–21 FoV were analyzed in any experimental condition. The number of synapses was calculated in 100-µm-long dendrites.

### Analysis of dendritic spine density

Primary hippocampal neurons were transfected at DIV5 with pcDNA3.1-mGreenLantern plasmid (Addgene, Cat. No. 161912) and Lipofectamine™ 3000 Transfection Reagent (Thermo Fisher Scientific, Cat. No. L3000015) according to the manufacturer’s instructions. Lipofectamine was removed 1 h after transfection to avoid cytotoxic effects on primary neurons. After fixation with 4% paraformaldehyde neurons were subjected to immunostaining with an anti-GFP antibody (Abcam, ab13970, 1:500) and the presence of mGreenLantern + neurons was assessed by means of IN Cell Analyzer 6000. Dendritic spine analysis was performed manually with Fiji (ImageJ; https://imagej.net/software/fiji/downloads) in blind for genotypes and treatments. Spines were classified according to criteria described in [23]. Spines were judged thin if their length was greater than the neck diameter. Spines were judged mushroom if the diameter of the head was much greater than the diameter of the neck. Spines were judged stubby if the diameter of the neck was similar to the total length of the spine. Number of spines was calculated in 100-µm-long dendrites. For each experimental condition 4–5 dendrites were analyzed. Since n = 3 biological replicates were performed a total of 12–15 dendrites were analyzed in any experimental condition.

### Induction of chemical LTP and electrophysiological recording

Whole-cell patch-clamp recordings were performed in voltage-clamp configuration at RT. Pipettes were prepared from borosilicate glass using a horizontal puller (P-97-Sutter Instruments) and, to isolate spontaneous excitatory postsynaptic currents (sEPSCs), they were filled with an intracellular solution containing 100 mM cesium methansulfonate, 25 mM CsCl, 2 mM MgCl_2_, 0.4 mM EGTA, 10 mM HEPES, 10 mM creatine phosphate, 0.4 mM Na-GTP (pH 7.4 with CsOH). The extracellular solution contained 125 mM NaCl, 1 mM MgCl_2_, 2 mM CaCl_2_, 2.5 mM KCl, 33 mM glucose, 5 mM HEPES, 0.02 mM bicuculline to block GABA_A_ receptors (pH 7.3 with NaOH). Pipette series resistance was constantly monitored during experiments. Spontaneous post-synaptic currents were recorded using an Axopatch 200B amplifier (Axon Instruments) and digitized with a Digidata 1322A AD/DA converter (Axon Instruments). Signals were acquired using Clampex software (Molecular Devices), sampled at 20–50 kHz, and low-pass filtered at 10 kHz using Clampfit 10.2 (Molecular Devices). Chemical LTP (cLTP) was induced by replacing, for a period of 15 min at RT, the external solution with a Mg-free “cLTP inducing solution” containing 125 mM NaCl, 2 mM CaCl_2_, 2.5 mM KCl, 33 mM glucose, 5 mM HEPES, 0.2 mM glycine, and 0.02 mM bicuculline (pH 7.3 with NaOH). Recordings of sEPSCs were performed after switching back to the regular extracellular solution. Treatments with the ADAM10 inhibitor GI254023X 1 µM or with the combination of GI254023X 1 µM and the TrkB antagonist ANA12 10 µM were performed by adding them to the medium and to the conditioning and recording solutions.

### Statistical analyses

Data are presented as means ± standard error of the mean (SEM) and were analyzed using GraphPad Prism Version 9.4.0 (453). For each data set we determined whether the data were normally distributed or not to select parametric or non-parametric statistical tests. Differences were considered statistically significant at P < 0.05. The specific statistical test used is indicated in the figure legends.

## Results

### ADAM10 active form is increased in the HD hippocampus

**Fig. 1:**
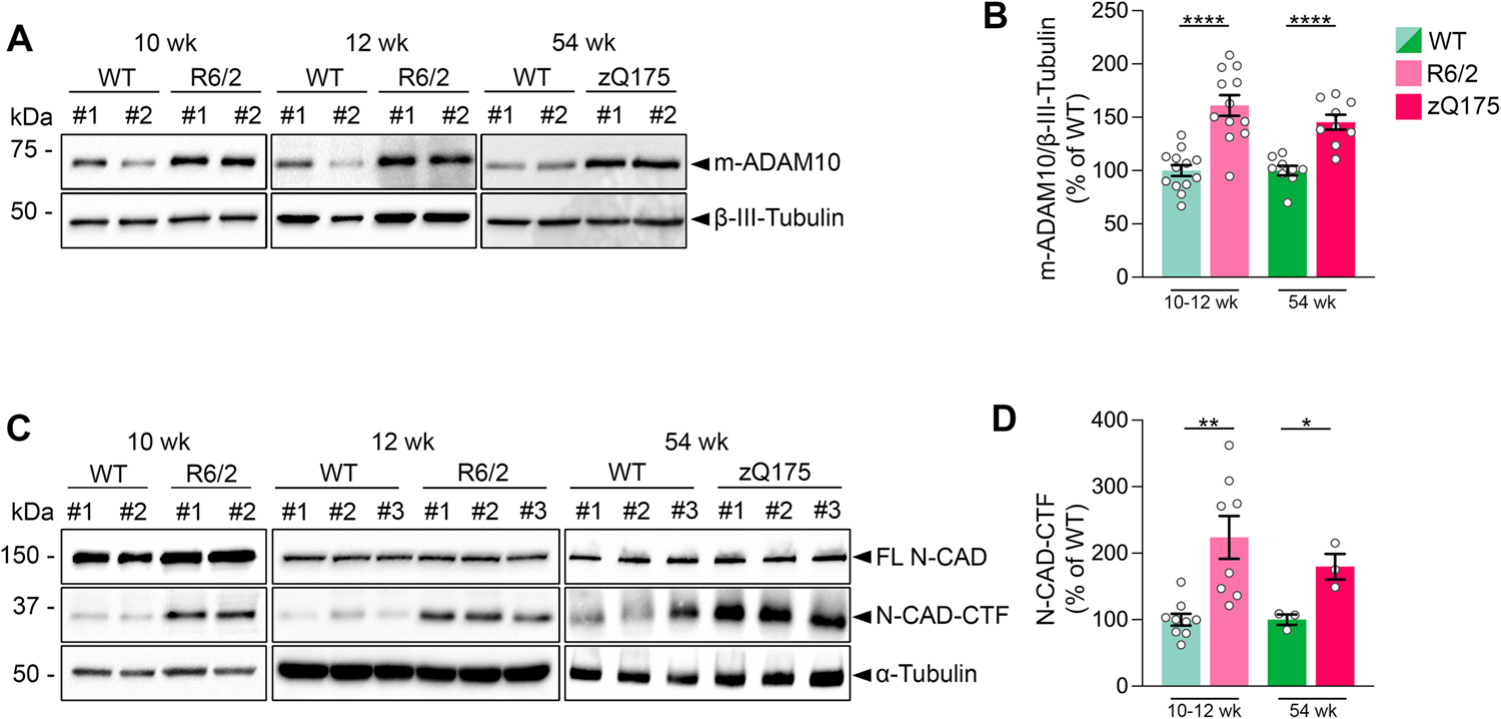
The mature active form of ADAM10 is increased in the HD mouse hippocampus and causes N-CAD proteolysis. **A** Representative Western blot for the mature active form of ADAM10 (m-ADAM10) in synaptosomal fractions obtained from the hippocampus of R6/2 transgenic mice and zQ175 heterozygous knock-in mice. β-III Tubulin, loading control. **B** Quantification of data shown in A. WT and R6/2 mice at 10–12 weeks of age:  n=12–13 mice/genotype. WT and zQ175 mice at 54 weeks of age: n=9 mice/genotype. Data are represented as mean ± SEM. ****P < 0.0001, unpaired t test. **C** Representative Western blot of N-CAD-CTF in the hippocampus from WT and HD mice (R6/2 and zQ175). α-Tubulin, loading control. **D** Quantification of results shown in C. The N-CAD-CTF signal intensity has been divided for the FL N-CAD content, which has been determined by dividing FL N-CAD intensity over the α-Tubulin intensity. WT and R6/2 mice at 10–12 weeks of age: n=8–9 mice/genotype; WT and zQ175 mice at 54 weeks: n=3 mice/genotype. Data are represented as mean ± SEM. *P < 0.05, **P < 0.01, unpaired t test

To evaluate the impact of ADAM10 dysfunction on the HD hippocampus, we first assessed the level of active ADAM10 in synaptosomal fractions prepared from the hippocampus of two HD mouse models. Initially, we focused on R6/2 transgenic mice, characterized by the presence of the exon 1 of the human HD gene with 144–150 CAG repeats under the control of the human HTT promoter, as described in [24]. These mice exhibit marked alterations in hippocampal synaptic plasticity and onset of cognitive and memory symptoms at 9 weeks of age, as described in [7, 18, 25]. The severity of these symptoms increases by 10–12 weeks, ultimately leading to death between 14 to 16 weeks of age [7, 18, 25, 26]. Western blot analysis on hippocampal synaptosomal fractions, performed with an antibody able to detect the mature active form of ADAM10, revealed a 61.1% increase in m-ADAM10 in symptomatic 10–12-week-old R6/2 mice compared to age-matched wild-type mice (Fig. 1A, B). Meanwhile, RT-qPCR did not reveal differences in the ADAM10 mRNA level between R6/2 and wild-type mice, indicating that the observed increase in hippocampal m-ADAM10 level in 10–12-week-old R6/2 mice was not due to increased ADAM10 transcription (Supplementary Fig. 1). Western blot analyses on isolated hippocampal sub-regions from 12-week-old wild-type mice showed that m-ADAM10 reaches the highest level in the Dentate Gyrus (DG) but the increase in m-ADAM10 level in the R6/2 hippocampus was restricted to the CA1 and CA3 sub-regions (Supplementary Fig. 2). We also show a 45.4% increase in m-ADAM10 in the hippocampus of 54-week-old symptomatic zQ175 mice, which offer an HD phenotype with heterozygous CAG expansion that recapitulates more closely the human condition [27] (Fig. 1A, B).

ADAM10 exerts its proteolytic action on a large repertoire of trans-synaptic proteins, including N-CAD which has a widely recognized role in hippocampal spine morphology and synaptic plasticity [28]. Increased N-CAD cleavage has already been shown in total lysates from HD striatum and cortex [18]. Here we tested whether higher synaptic m-ADAM10 content in synaptosomal fractions resulted in enhanced N-CAD shedding in the HD hippocampus. We monitored ADAM10-mediated N-CAD cleavage by quantifying the ~ 36 kDa C-terminal fragment of N-CAD (N-CAD-CTF) [29]. We found that the level of N-CAD-CTF was 123.8% higher in the hippocampus of 10–12-week-old symptomatic R6/2 mice compared to age-matched wild-type mice (Fig. 1C, D). Similar results were obtained in the hippocampus of heterozygous 54-week-old zQ175 mice, which showed a 79.7% increase in N-CAD-CTF when compared to controls (Fig. 1C, D).

Taken together, these results indicate that mutant HTT causes an accumulation of active ADAM10 in the HD hippocampal synapse, which correlates with increased N-CAD proteolysis.

### Normalization of the active ADAM10 level prevents the loss of spines in the R6/2 CA1 region

**Fig. 2:**
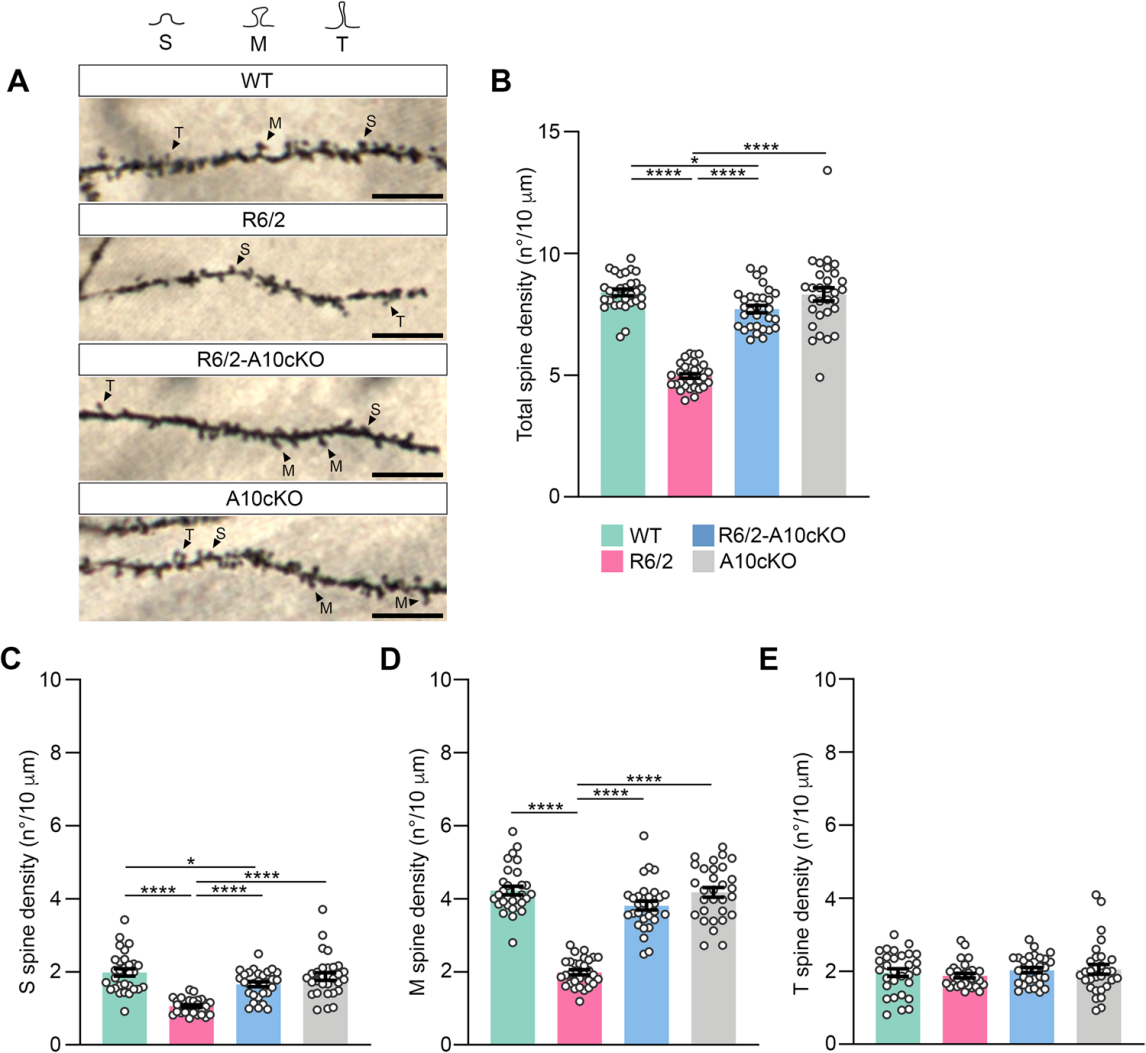
ADAM10 heterozygous deletion in the forebrain rescues dendritic spine loss in the CA1 region of the hippocampus in R6/2 mice. **A** Representative examples of secondary apical dendritic segments of CA1 pyramidal neurons from 13-week-old WT, R6/2, R6/2-A10cKO and A10cKO mice. Scale bars: 10 µm, 80 × Objective. S, stubby spines; M, mushroom spines; T, thin spines. **B** Total dendritic spine density. **C** Stubby spine density. **D** Mushroom spine density. **E** Thin spine density. In B-E n = 3 mice/genotype were analyzed for a total of n = 30 neurons/genotype. Each dot in the graphs represents the mean ± SEM of the spine density in 10 µm dendrite for each neuron analyzed. *P < 0.05, ****P < 0.0001, One-way ANOVA with Tukey’s post hoc test

Dendritic spine pathology has been described in the hippocampus of HD mice, including the R6/2 transgenic mouse model [30]. To explore whether reduced spine density in the R6/2 hippocampus is related to the observed increase in m-ADAM10, we crossed R6/2 mice with *CaMKIIα-Cre:Adam10*^*Flox/+*^ mice (A10cKO), an ADAM10 heterozygous conditional knock-out (KO) mouse line restricting ADAM10 gene inactivation to the postnatal forebrain, to generate R6/2-A10cKO mice [18]. Supplementary Fig. 3 shows that m-ADAM10 level was reduced close to the wild-type level in the hippocampus of 13-week-old R6/2-A10cKO mice. Next, we evaluated the number of spines on Golgi-stained dendrites from 13-week-old wild-type, R6/2, R6/2-A10cKO, and A10cKO mice. Representative images of dendritic spines in the CA1 region of wild-type, R6/2, R6/2-A10cKO, and A10cKO mice are shown in Fig. 2A. Consistent with previous reports [30], R6/2 mice exhibited decreased spine density—defined as the total of all spines, including those with small or immature synapses such as thin (T) spines, and those with mature and large synaptic contacts such as mushroom (M) and stubby (S) spines—in CA1 apical dendrites compared to wild-type mice (Fig. 2A, B). Importantly, normalization of active ADAM10 level in R6/2-A10cKO mice alleviated dendritic spine loss in CA1 pyramidal neurons (Fig. 2A, B). Total dendritic spine density, as well as density of S, M and T spines in CA1 hippocampal neurons of A10cKO mice were not significantly different from that of age-matched wild-type littermates (Fig. 2A–E), indicating that heterozygous deletion of the ADAM10 gene does not induce changes in spine density and morphology. We also show that the loss of spines in the CA1 of 13-week-old R6/2 mice can be attributed to a decrease in spines with mature and large synaptic contacts, such as S and M spines (Fig. 2C, D), while dynamic T spines were not affected by the HD mutation (Fig. 2E). Notably, we found that the density of spines that form strong and stable synaptic connections was restored by active ADAM10 normalization in R6/2-A10cKO mice (Fig. 2C, D).

Collectively, these findings underscore the impact of genetically reducing ADAM10 in preventing enduring loss of dendritic spines in hippocampal neurons affected by HD.

### Normalization of the active ADAM10 level prevents ultrastructural defects in the HD hippocampal synapse

**Fig. 3:**
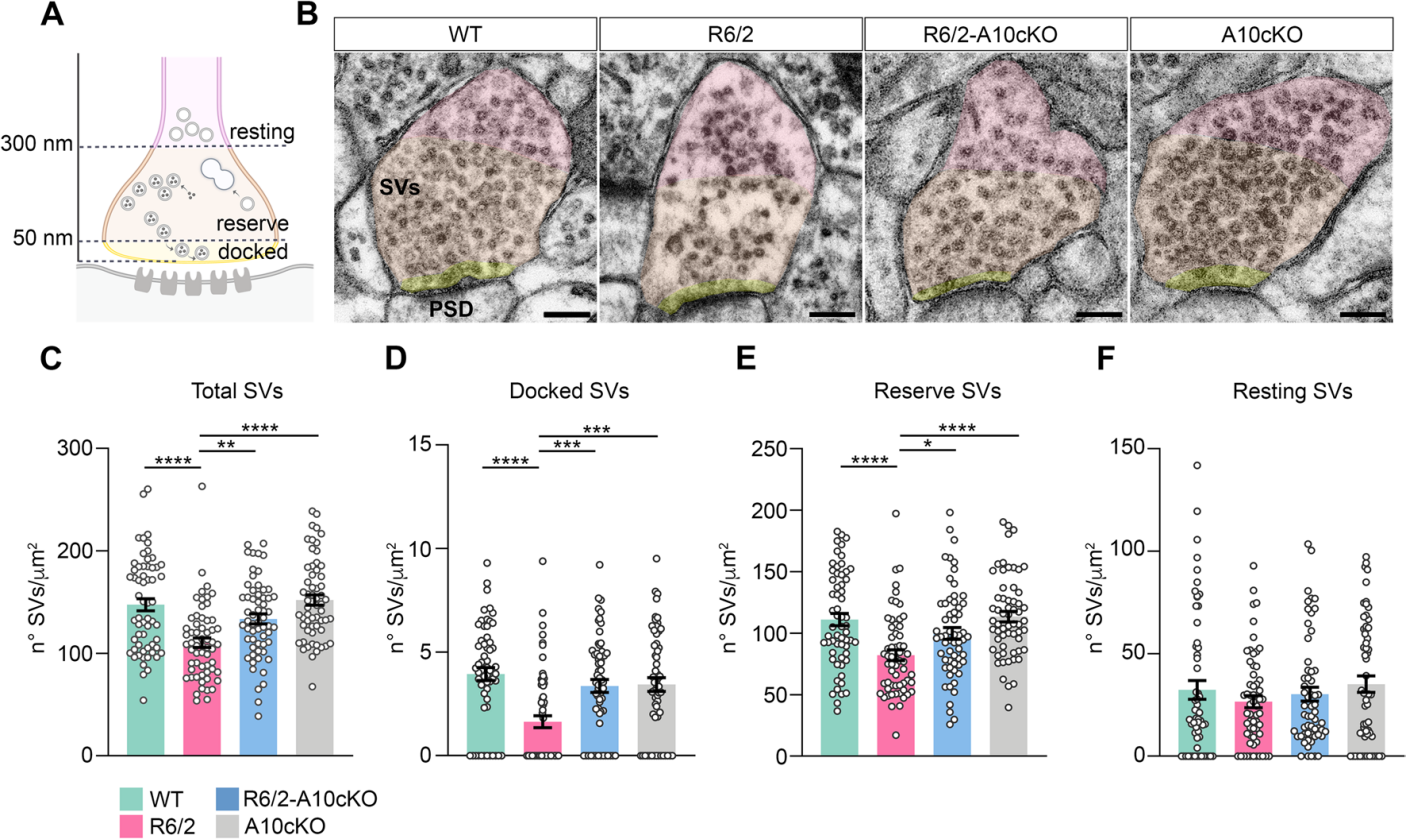
ADAM10 heterozygous deletion in the forebrain rescues ultrastructural defects of the HD hippocampal synapse. **A** Diagram showing SVs classification based on distance from the presynaptic membrane (docked: 0–50 nm, reserve: 50–300 nm, resting: > 300 nm) with corresponding tenuous background colors added as a guide for the eye in TEM images reported in panel (**B**). **B** Representative TEM images of excitatory synapses in pyramidal neurons of the CA1 region of the hippocampus of WT, R6/2, R6/2-A10cKO and A10cKO mice at 13 weeks of age. Scale bars: 100 nm. PSD, post-synaptic density. **C** Density of total SVs. **D** Density of docked SVs. **E** Density of reserve SVs. **F** Density of resting SVs. In C-F, n = 3 mice/genotype and n = 60 excitatory synapses/genotype were analyzed. Each dot in the graphs represents the n° SVs/µm^2^ for each excitatory synapse analyzed. Data are presented as mean ± SEM. *P < 0.05, **P < 0.01, ***P < 0.001, ****P < 0.0001, Kruskal–Wallis with Dunn’s multiple comparisons test

Next, we performed transmission electron microscopy (TEM) imaging to characterize the ultrastructural morphology of the excitatory synapse in hippocampal CA1 neurons of wild-type and R6/2 mice. We first examined SVs density in the presynapse (Fig. 3A, B). As expected, SVs density was reduced in CA1 presynaptic boutons of R6/2 mice compared to wild-type mice (Fig. 3B, C). Among the SVs, the docked SVs are connected to the presynaptic membrane and rapidly fuse in response to a presynaptic action potential, therefore forming the readily releasable pool (RRP) [31] (Fig. 3A). We found that the density of these docked SVs, identified within 50 nm of the active zone, was significantly reduced in the R6/2-CA1 bouton (Fig. 3B, D). SVs density in the reserve pool (RP), which includes vesicles identified between 50 and 300 nm from the active zone as a source to refill the RRP after exocytosis (Fig. 3A), was also reduced in CA1-R6/2 boutons (Fig. 3B, E). In contrast, there  were no variations between genotypes in the density of SVs located beyond 300 nm from the active zone and termed resting SVs, which participate in exocytosis only after sustained stimulation [31] (Fig. 3B, F). We concluded that mutant HTT reduces SVs density in those pools with a higher turnover and actively engaged in SVs release and recycling. On the postsynaptic side, we measured the postsynaptic density (PSD) length, a parameter that can be affected by changes in synaptic efficacy and by altered plasticity [32]. We found that the PSD length was smaller in the R6/2 hippocampal CA1 pyramidal neurons compared to wild-type neurons, which reflects reduced anchoring of receptors and adaptor proteins orchestrating synaptic signaling (Supplementary Fig. 4). Notably, the observed pre- and postsynaptic defects were rescued in the R6/2-A10cKO hippocampus (Fig. 3B–E; Supplementary Fig. 4). No alterations in morphological pre- and postsynaptic parameters were identified in A10cKO mice, indicating that heterozygous deletion of the ADAM10 gene in the wild-type hippocampus had no influence on the ultrastructural morphology of the excitatory synapse (Fig. 3B–F; Supplementary Fig. 4).

All together, these data show the existence of presynaptic and postsynaptic pathology at the excitatory synapse in the CA1 region of the R6/2 hippocampus, which can be reversed by normalizing the active ADAM10 level.

### Inhibiting ADAM10 activity increases synaptic BDNF and activates ERK in the HD hippocampus

The loss of BDNF mRNA and protein has been widely documented in HD [33–36]. The BDNF/TrkB pathway is also recognized for its role in regulating dendritic spine density and morphology in the hippocampus, as well as inducing LTP [37]. Additionally, a decrease in BDNF content and TrkB phosphorylation has been associated with spine loss and LTP defects in HD mouse models, including R6/2 mice [10, 14, 38, 39]. Based on these findings, we further investigated whether normalizing ADAM10 could mitigate BDNF loss in the hippocampus of R6/2 mice.

**Fig. 4:**
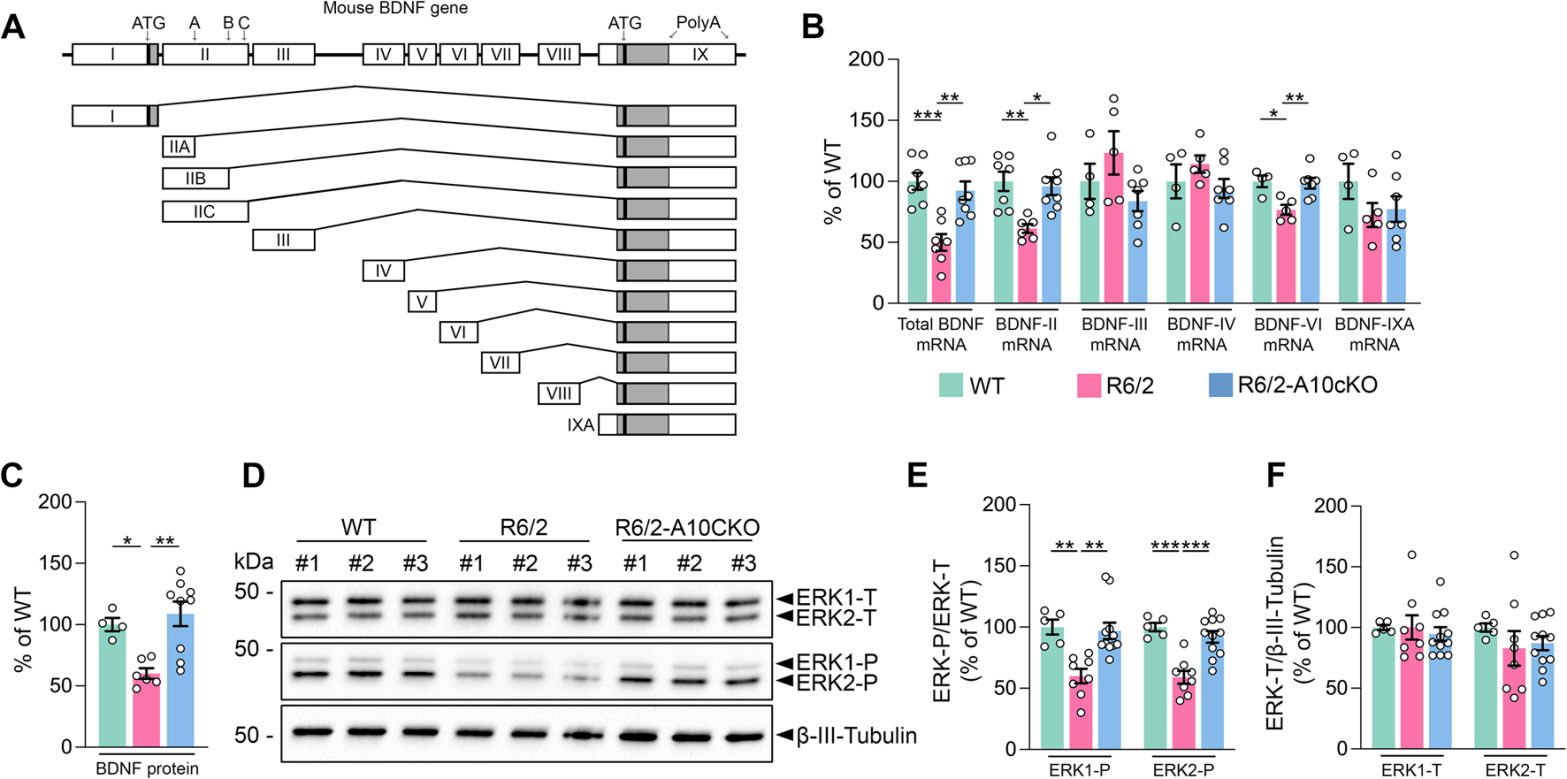
ADAM10 heterozygous deletion in the forebrain enhances BDNF synthesis and promotes ERK phosphorylation in the R6/2 hippocampus. **A** Scheme of the mouse BDNF gene and BDNF mRNA isoforms. **B** Total BDNF mRNA level and level of BDNF mRNA isoforms in the hippocampus of WT, R6/2 and R6/2-A10cKO mice at 13 weeks of age. WT: n = 4–7; R6/2: n = 5–7; R6/2-A10cKO: n = 7–8. Data are represented as mean ± SEM. *P < 0.05, **P < 0.01, ***P < 0.001, One-way ANOVA with Bonferroni’s post hoc test. For BDNF mRNA isoform II the forward and reverse primers (see Methods) led to simultaneous amplification of the transcript variant IIA, IIB, and IIC. **C** ELISA for BDNF in the hippocampus of WT, R6/2 and R6/2-A10cKO mice at 13 weeks of age. WT: n = 4; R6/2: n = 6; R6/2-A10cKO: n = 9. Data are represented as mean ± SEM. *P < 0.05, **P < 0.01, One-way ANOVA with Bonferroni’s post hoc test. **D** Representative Western blot for total and phosphorylated ERK1/2 in the hippocampus of WT, R6/2 and R6/2-A10cKO mice at 13 weeks of age. β-III Tubulin, loading control. **E**, **F** Quantification of data in D. WT: n = 5; R6/2: n = 8; R6/2-A10cKO: n = 11. Data are represented as mean ± SEM. **P < 0.01, ***P < 0.001, One-way ANOVA with Bonferroni’s post hoc test

The transcription of the *Bdnf* gene in rodents is regulated by multiple promoters that enable complex cell-type and stimulus-specific *Bdnf* expression [40]. Eight noncoding 5′ exons (I–VIII) are alternatively spliced to the 3′ protein-encoding exon IX to produce eight BDNF isoforms in rodents [40] (Fig. 4A). Moreover, transcription can be initiated in the intron before the protein coding exon, which results in IXA transcript containing the 5′ extended coding exon [40]. Using RT-qPCR and primers targeting specifically the 3’-UTR region, which is shared by all BDNF mRNA isoforms, we found that total BDNF mRNA content was 50.1% decreased in the hippocampus of 12-week-old R6/2 mice compared with age-matched controls (Fig. 4B). Notably, total BDNF mRNA was significantly increased in the R6/2-A10cKO hippocampus and was almost comparable to the level reported in wild-type mice (Fig. 4B). Previous studies indicated that mutant HTT downregulates the activity of BDNF promoters II, IV and VI in the HD brain [33, 34, 41, 42]. As shown in Fig. 4B, the levels of BDNF mRNA II and VI were considerably lower in the hippocampus of 12-week-old R6/2 mice compared to wild-type mice. The other BDNF mRNA isoforms were barely detectable or unaffected by the HD mutation (Fig. 4B). Notably, levels of BDNF mRNA II and VI were restored in the R6/2-A10cKO hippocampus (Fig. 4B). By employing an ELISA assay on synaptosomal fractions, we also found that BDNF synaptic level was reduced by 40% in the R6/2 hippocampal tissues compared to wild-type and restored in R6/2-A10cKO mice (Fig. 4C). These data suggest that normalization of the active ADAM10 level restores the synaptic BDNF content in the HD hippocampus.

Binding of BDNF to its receptor TrkB activates the Extracellular signal-regulated kinase 1/2 (ERK1/2) phosphorylation, which is critical for dendritic spine formation in hippocampal neurons [43]. We showed that ERK1-P and ERK2-P levels were reduced by  40.1% and 41.0%, respectively, in the hippocampus of 12-week-old R6/2 mice compared to wild-type mice. Conversely, hippocampal ERK1-P and ERK2-P levels reverted close to the wild-type level in R6/2-A10cKO mice (Fig. 4D, E). The levels of total ERK1 (ERK1-T) and total ERK2 (ERK2-T) were not statistically different between the three analyzed genotypes (Fig. 4D, F). We also investigated whether an acute paradigm of ADAM10 inhibition in vivo could be effective in enhancing neuroprotective ERK1/2 phosphorylation in symptomatic R6/2 mice. To this end, we employed symptomatic 12-week-old R6/2 mice and TAT-Pro-ADAM10^709–729^ cell penetrating peptides designed to block the enzyme trafficking to the membrane [44]. Specifically, TAT-Pro-ADAM10^709–729^ contains the poly-proline stretch of ADAM10 required for binding to SAP97 and, by sequestering SAP97, it blocks SAP97-mediated ADAM10 trafficking to the synapse and its activity [18, 44]. As control, the analogous inactive peptide TAT-Ala-ADAM10^709–729^ in which all proline residues were substituted by alanines was used [44]. Twelve-week-old R6/2 mice received 2 intraperitoneal injections 24 h apart of either TAT-Pro-ADAM10^709–729^ or TAT-Ala-ADAM10^709–729^. Our data showed that acute exposure to TAT-Pro-ADAM10^709–729^ increased ERK1-P and ERK2-P in symptomatic R6/2 mice, while the control peptide (TAT-Ala-ADAM10^709–729^) had no effect (Supplementary Fig. 5).

Taken together, these data indicate that normalizing mature ADAM10 level leads to an increase in BDNF gene transcription, the pool of synaptic BDNF protein, and ERK neuroprotective signaling in the HD hippocampus.

### Neuroprotection through ADAM10 inhibition requires TrkB

**Fig. 5:**
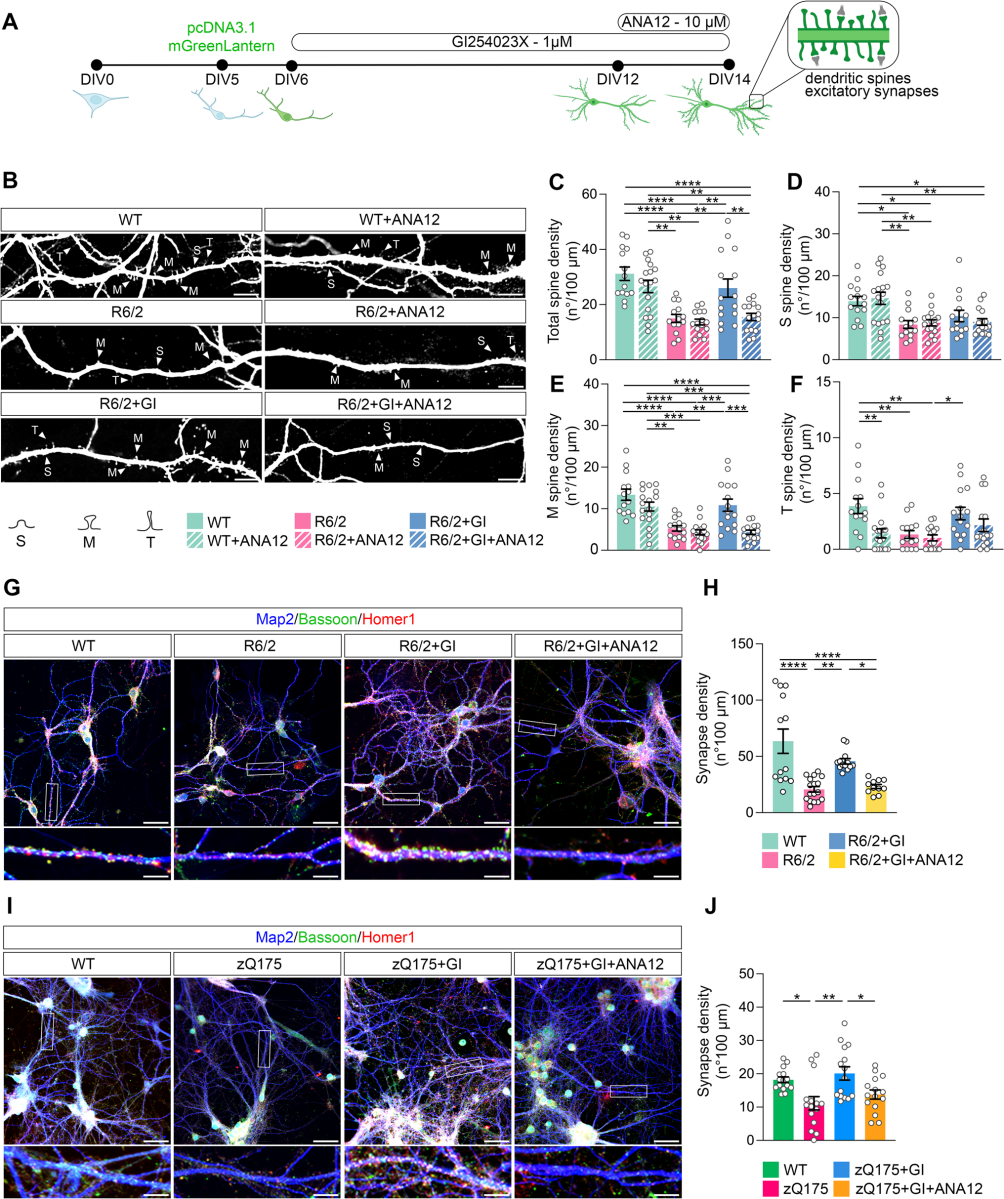
TrkB mediates the neuroprotective effect determined by ADAM10 inhibition on long-lasting spine loss in HD hippocampal neurons. **A** Hippocampal neurons from WT and R6/2 mice were transfected at DIV5 with pcDNA3.1-mGreenLantern plasmid. The ADAM10 inhibitor GI254023X (GI, 1 µM) was administered from DIV6 until DIV14. The TrkB antagonist ANA12 (10 µM) was administered at DIV12 and cells were fixed at DIV14 for spine analyses and excitatory synapses quantification. **B** Immunofluorescence images of dendritic spines in hippocampal cultures: WT, WT + ANA12, R6/2, R6/2 + ANA12, R6/2 + GI; R6/2 + GI + ANA12. Scale bars: 10 µm. M, mushroom spines; T, thin spines; S, stubby spines. **C-F** Density of total, stubby, mushroom, and thin spines. Data are from n = 3 independent primary culture preparations. Each dot in the graphs represents the number of spines in a 100-µm-long dendrite. Data are presented as mean ± SEM. *P < 0.05, **P < 0.01, ***P < 0.001, ****P < 0.0001, One-way ANOVA with Tukey’s post hoc test. **G** Immunofluorescence images of excitatory synapses in hippocampal cultures: WT, R6/2, R6/2 + GI; R6/2 + GI + ANA12. **I** Immunofluorescence images of excitatory synapses in hippocampal cultures: WT, zQ175, zQ175 + GI; zQ175 + GI + ANA12. Excitatory synapses in G and I were visualized by Bassoon/Homer1 immunostaining. Map2, pan neuronal marker. Upper panel scale bars: 50 μm; bottom panel scale bars: 10 μm. **H**, **J** Synapses quantification. Data are from n = 3 independent primary culture preparations. Each dot in the graphs represents the number of excitatory synapses in a 100-µm-long dendrite. Data are presented as mean ± SEM. *P < 0.05, **P < 0.01, ****P < 0.0001, One-way ANOVA with Tukey’s post-hoc test

As ADAM10 inhibition increases the level of BDNF in the hippocampus of R6/2 mice, we tested whether the resulting neuroprotection occurs through TrkB activation. To do so, we treated primary hippocampal neurons from wild-type and R6/2 mice with both the ADAM10 pharmacological inhibitor GI254023X [45, 46] and the TrkB antagonist ANA12 [47]. GI254023X belongs to the hydroxamate class of metallopeptidase inhibitors and selectively inhibits ADAM10 by chelating the zinc ion in its catalytic site [18, 45, 46]. ANA12 is a low-molecular-weight heterocyclic compound that, by binding to the extracellular domain of TrkB, prevents BDNF-induced TrkB activation, and abolishes the biological effects of BDNF on TrkB-expressing cells [47]. Primary hippocampal cultures were prepared from E18 wild-type and R6/2 fetuses and transfected on DIV5 with a plasmid encoding mGreenLantern for visualization of dendritic spines. According to the experimental scheme described in Fig. 5A, R6/2 cultures were treated with GI254023X 1 µM every 48 h from DIV6 until DIV14. Treatment with the TrkB antagonist ANA12 10 µM was performed from DIV12 to DIV14. Cultures were then fixed at DIV14 for imaging of dendritic spines and excitatory synapses (Fig. 4A). We found that ANA12 treatment alone reduced the density of thin spines in wild-type hippocampal neurons (Fig. 5B, F). Nonetheless, because thin spines at DIV14 represent less than 13% of total spines, the density of total dendritic spines remained unchanged in the wild-type culture treated with ANA12 (Fig. 5B–C). We also found that R6/2 hippocampal neurons in vitro exhibited reduced total spine density (Fig. 5B–C) due to loss of stubby, mushroom, and thin spines (Fig. 5D–F). Treatment with 10 µM ANA12 for 48 h from DIV12 to DIV14 did not induce any change in spine density in R6/2 hippocampal neurons (Fig. 5B–F). Notably, we reported that eight days of treatment with GI254023X 1 µM reduced dendritic spine loss in the R6/2 hippocampal neurons (Fig. 5B, C). In particular, ADAM10 inhibition prevented mushroom spines loss (Fig. 5B, E) but this beneficial effect was significantly reduced when hippocampal R6/2 neurons also received the TrkB antagonist ANA12 (Fig. 5B, E). These data indicate that mushroom spine loss recovery in the HD hippocampus through ADAM10 inhibition requires an active TrkB.

Since mushroom spines are the site of mature and functional excitatory synapses [48], we performed immunocytochemistry for Bassoon and Homer1, which label the pre- and postsynaptic terminals of the excitatory synapse, respectively [49]. By counting the number of Bassoon + /Homer1 + co-localizing puncta, we showed a significant reduction in the number of total synapses in R6/2 hippocampal neurons compared to wild-type neurons (Fig. 5G, H). GI254023X treatment, according to the experimental scheme in Fig. 5A, increased Bassoon + /Homer1 + co-localizing puncta in DIV14 R6/2 hippocampal primary neurons, but this effect was not seen in the presence of the TrkB antagonist ANA12 (Fig. 5G, H). Similar results were observed when the experimental paradigm in Fig. 5A was applied to hippocampal neurons generated from zQ175 heterozygous mutant HTT knock-in mice (Fig. 5I, J).

Overall, these results indicate that BDNF-induced TrkB activation contributes to mediate the neuroprotective effect determined by ADAM10 inhibition on long-lasting spine loss in HD hippocampal neurons.

### Inhibiting ADAM10 induces LTP and formation of new spines in HD hippocampal neurons via TrkB

**Fig. 6:**
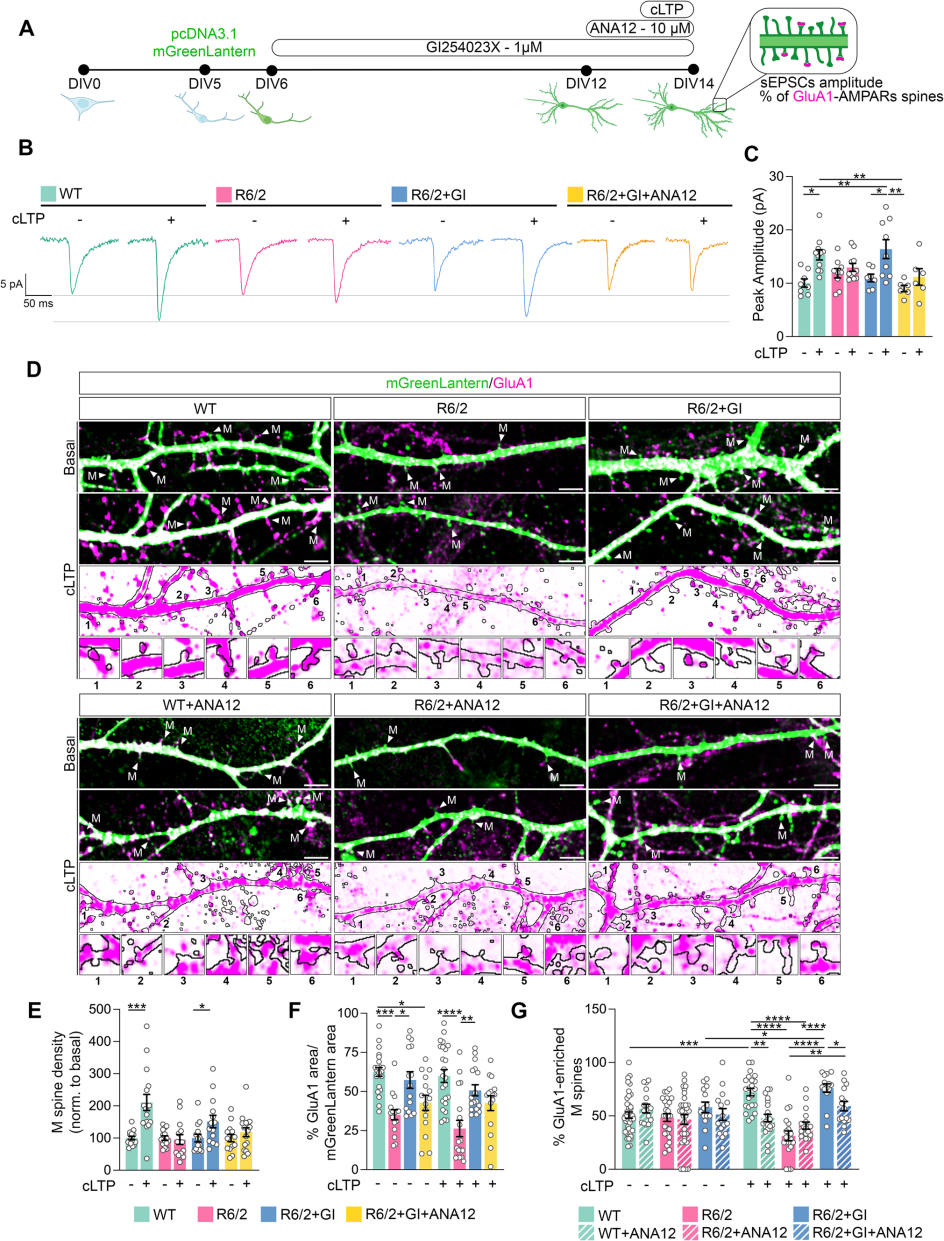
Blocking active ADAM10 with GI254023X promotes LTP induction through the TrkB signaling pathway. **A** Experimental scheme of treatment of WT and R6/2 primary hippocampal neurons. The ADAM10 inhibitor GI254023X (GI, 1 µM) was administered from DIV6 until DIV14. The TrkB antagonist ANA12 (10 µM) was administered at DIV12 until DIV14. Chemical LTP was induced at DIV14 with 0.2 mM glycine for 15 min. For dendritic spine analyses hippocampal neurons were transfected at DIV5 with pcDNA3.1-mGreenLantern plasmid. **B** Representative traces of spontaneous EPSCs (sEPSCs) recorded at a holding potential of -70 mV in baseline condition and following chemical LTP-induction (cLTP) in primary hippocampal cell cultures obtained from WT and R6/2 mice. + GI and + ANA12 indicate the presence of these substances in culture medium and during electrophysiological recordings. **C** Graph comparing the amplitudes of sEPSCs in baseline condition and after cLTP induction in the different experimental conditions. Each dot corresponds to the value obtained from a single cell. Data are expressed as mean ± SEM and were analyzed by Two-way ANOVA with Bonferroni’s post hoc test. *P < 0.05, **P < 0.01. **D** Representative images of dendritic segments (mGreenLantern signal) and GluA1 immunostaining in basal condition and after cLTP induction. M, mushroom spines. Image crops of representative M spines were numbered from 1 to 6. Scale bars: 10 µm. **E** Quantification of mushroom spine density. Data are from n = 3 independent primary culture preparations. Each dot in the graph represents the number of mushroom spines in a 100-µm-long dendrite. Data are shown as % over the basal condition, which was set to 100, and are expressed as mean ± SEM. *P < 0.05, ***P < 0.001, unpaired t test. **F** Quantification of GluA1 signal. Data are from n = 3 independent primary culture preparations. Each dot in the graph represents GluA1 signal in a 100-µm-long dendrite. Data are expressed as mean ± SEM and were analyzed by Two-way ANOVA with Tukey’s post hoc test. P* < 0.05, **P < 0.01, ***P < 0.001, ****P < 0.0001. **G** Percentage of mushroom spines enriched in GluA1 in basal condition and after cLTP induction. Data are from n = 3–5 independent primary culture preparations. Each dot in the graph represents the number of mushroom spines in a 100-µm-long dendrite. Data are expressed as mean ± SEM and statistical analysis was performed by using Two-way ANOVA with Tukey’s post hoc test. P* < 0.05, **P < 0.01, ***P < 0.001, ****P < 0.0001. Supplementary Table 1 and 2: detailed statistical outputs related to panel F and G

Given that both in vitro and in vivo paradigms of ADAM10 inhibition have successfully restored the density of long-lasting spines in HD hippocampal neurons, we sought to determine whether interfering with ADAM10 activity could induce NMDAR-dependent LTP. Notably, this form of LTP is severely compromised in the HD hippocampus, as evidenced by previous studies [7, 10, 11]. We therefore induced NMDAR-dependent synaptic plasticity in hippocampal cultures using a chemical LTP (cLTP) protocol, which consists in bath application of glycine (0.2 mM for 15 min, in a Mg^2+^-free solution), a co-agonist of the glutamatergic NMDAR [50]. This protocol shares biochemical, morphological, and functional synaptic responses with NMDA-dependent LTP in the hippocampus in vivo [50]. Specifically, cLTP causes an increase in the amplitude of spontaneous excitatory post-synaptic currents (sEPSCs), leading to spine expansion and synapse formation [51]. Figure 6A shows the experimental design carried out to evaluate the effect of the ADAM10 inhibitor GI254023X on LTP defects in R6/2 hippocampal neurons. Membrane capacitance, a parameter that correlates with the overall dimension of the cell, and changes in sEPSCs were examined in basal condition and after cLTP induction in the following four groups: WT, R6/2, R6/2 + GI254023X, and R6/2 + GI254023X + ANA12. Data were analyzed by considering as independent variables both the four conditions and the cLTP induction. We found no differences in membrane capacitance, sEPSCs rise time, or sEPSCs decay time between the groups before and after cLTP induction (Supplementary Fig. 6). No differences were present in sEPSCs amplitude before cLTP induction in the four analyzed groups (Fig. 6B, C). Conversely, a significant increase in sEPSCs amplitude following cLTP induction was observed in wild-type, but not in R6/2 hippocampal neurons (Fig. 6B, C), indicating that cLTP was successfully induced in wild-type, but not in mutant HTT expressing neurons. These data indicate that our in vitro paradigm for cLTP induction recapitulates LTP defects that are documented in vivo in the HD hippocampus [7, 10, 11]. Noteworthy, when the ADAM10 inhibitor GI254023X was applied to R6/2 hippocampal neurons, the amplitude of sEPSCs was significantly increased after cLTP stimulation compared to the basal condition (Fig. 6B, C). These findings indicate that ADAM10 inhibition effectively restores NMDAR-dependent LTP in R6/2 hippocampal neurons. NMDAR-dependent LTP requires the simultaneous fulfillment of several conditions to occur, one of which is the activation of the BDNF/TrkB pathway [37]. Therefore, impairments of the BDNF/TrkB pathway block NMDA-dependent LTP induction in HD [15]. Consistently, we found that cLTP was not induced in R6/2 hippocampal neurons when we co-administered GI254023X and ANA12 (Fig. 6B, C). Since LTP induction is unlikely to happen in the presence of a persistent deficit in the BDNF pathway, we concluded that TrkB signaling participates in the GI254023X-induced restoration of LTP in R6/2 hippocampal neurons.

Since NMDAR-dependent cLTP is known to promote the formation of long-lasting spines [51], we quantified mushroom spine density after cLTP stimulation in each of the four experimental conditions that were examined. We revealed that cLTP stimulation by glycine increased mushroom spine density in wild-type but not in R6/2 hippocampal neurons (Fig. 6D, E). Additionally, spine density significantly increased in GI254023X-pretreated R6/2 hippocampal neurons upon cLTP induction (Fig. 6D, E). On the contrary, mushroom spine density was not increased in the R6/2 hippocampal culture co-treated with GI254023X and ANA12 after LTP induction (Fig. 6D, E).

We then evaluated GluA1 + mushroom spine density since GluA1-AMPARs subunit incorporation in these spines is crucial for both LTP and long-lasting spine formation in paradigms of NMDAR-dependent plasticity [50, 52, 53]. We showed that in unstimulated condition GluA1 subunit protein level was decreased in R6/2 hippocampal neurons compared to wild-type neurons (Fig. 6F). These data indicate that GluA1 protein level is hampered in the presence of mutant HTT. Conversely, GI254023X treatment increased GluA1 protein level in R6/2 hippocampal neurons (Fig. 6F). After cLTP induction GluA1 protein level did not increase in either wild-type or R6/2 cultures (Fig. 6F). This is expected since cells have been fixed for GluA1 immunocytochemical analyses 40 min after cLTP induction, and it is recognized that two to three hours are needed upon induction of LTP to induce new protein synthesis [54]. After cLTP induction, the percentage of GluA1 + mushroom spines was increased in wild-type hippocampal neurons, but ANA12 treatment alone reduced the percentage of GluA1 + mushroom spines in these neurons (Fig. 6G). This is expected due to the role of BDNF/TrkB pathway in GluA1-AMPARs trafficking to the PSD and in LTP induction [55]. We observed that the exposure to ANA12 did not further reduce the percentage of GluA1 + mushroom spine density in R6/2 cultures (Fig. 6G), because the BDNF/TrkB pathway is already significantly compromised [7, 15]. Notably, GI254023X treatment was beneficial to R6/2 neurons and increased the percentage of GluA1 + mushroom spines (Fig. 6G). This effect was partially lost when GI254023X and ANA12 were both administered to the R6/2 hippocampal neurons (Fig. 6G).

All together, these findings indicate that ADAM10 inhibition promotes NMDA-dependent LTP induction and the formation of mushroom spines enriched with GluA1-AMPARs in HD hippocampal neurons. We also show that ADAM10 inhibition requires a functional BDNF/TrkB pathway to promote LTP induction.

## Discussion

Our prior research highlighted the elevated level of active ADAM10 in the cortex and striatum as a crucial factor in the synaptic dysfunction and cognitive decline associated with HD [18, 19]. In this new study, we extend these findings by demonstrating an increased level of active ADAM10 and proteolysis of its synaptic target N-CAD in the hippocampus of two HD mouse models. These changes have detrimental effects on both structural and functional synaptic plasticity in the HD hippocampus. Our evidence indicates that the hyperactive ADAM10 impedes LTP and leads to a persistent loss of spines, concurrently inducing ultrastructural abnormalities within the glutamatergic synapse. Conversely, our findings show that conditional heterozygous deletion of ADAM10 in the forebrain of HD mice or its chemical inhibition with GI254023X effectively counteracts the biochemical, ultrastructural, and functional plasticity defects observed in the HD hippocampus.

According to numerous studies, ADAM10 regulates dendritic spines and LTP in hippocampal neurons through its proteolytic action on synaptic cell adhesion proteins N-CAD, Neuroligin 1, and Nectin 1 [17, 56]. One of the most interesting findings of this study is that the BDNF receptor TrkB emerges as a new effector through which ADAM10 inhibition exerts its neuroprotective effect in the HD hippocampus. The ability of the ADAM10 inhibitor GI254023X to induce LTP and to protect long-lasting spines was reduced in the presence of the TrkB antagonist ANA12. Our results indicate that ADAM10 acts upstream of the BDNF/TrkB pathway to regulate hippocampal synaptic plasticity. By inhibiting ADAM10, we enhance BDNF gene transcription, restore synaptic BDNF protein level, and boost ERK phosphorylation in the HD mouse hippocampus. These findings have important implications for the biology of the excitatory synapse, particularly the unexpected link discovered between ADAM10 and the TrkB pathway. More importantly, this discovery holds significance for HD treatment as inhibiting ADAM10 can prevent two critical molecular dysfunctions in the HD synapse: synaptic cell adhesion defects [18] and downregulation of the BDNF/TrkB pathway [14]. Both dysfunctions are crucial in the process leading to LTP defects and synapse loss in HD.

We demonstrate that normalizing active ADAM10 increases BDNF mRNA level in HD by activating transcription from BDNF promoters II and VI. This finding introduces a complex scenario of new mechanistic hypotheses. BDNF promoter II is regulated by the transcriptional repressor RE1-Silencing Transcription Factor/Neuron-Restrictive Silencer Factor (REST/NRSF) which binds to the Neuron-Restrictive Silencer Element (NRSE) within BDNF exon II [57]. Mutation of wild-type HTT results in loss of interaction with REST/NRSF, which accumulates in the nucleus leading to reduced transcription of BDNF exon II mRNA in HD [34, 36]. Several reports include the REST/NRSF among β-catenin target genes [58, 59]. ADAM10-induced N-CAD cleavage has a fundamental role in the redistribution of β-catenin from the cell surface pool to the cytoplasm, resulting in the activation of β-catenin target genes [29]. Along these lines, the most likely explanation is that the normalization of ADAM10 in HD reduces the cytoplasmic pool of β-catenin and REST/NRSF synthesis, thus increasing BDNF exon II mRNA in the HD hippocampus. Clarifying the molecular events and explaining how inhibition of ADAM10 increases the activity of BDNF promoter VI will be particularly challenging due to the interplay among the multiple transcription factors implicated in its regulation [60]. The observation that ADAM10 and HTT interactomes share presynaptic binding partners involved in axonal transport and in the regulation of synaptic vesicle homeostasis [19] is consistent with the hypothesis that strategies that normalize ADAM10 level in the HD brain may also facilitate the transport and release of growth factors and neurotransmitters. Future studies will be necessary to fully understand the molecular mechanisms and roles of ADAM10 in the biology of the BDNF/TrkB pathway. Despite these open questions, the findings obtained in this study are relevant in HD and increase interest in ADAM10 as a therapeutic target.

**Fig. 7:**
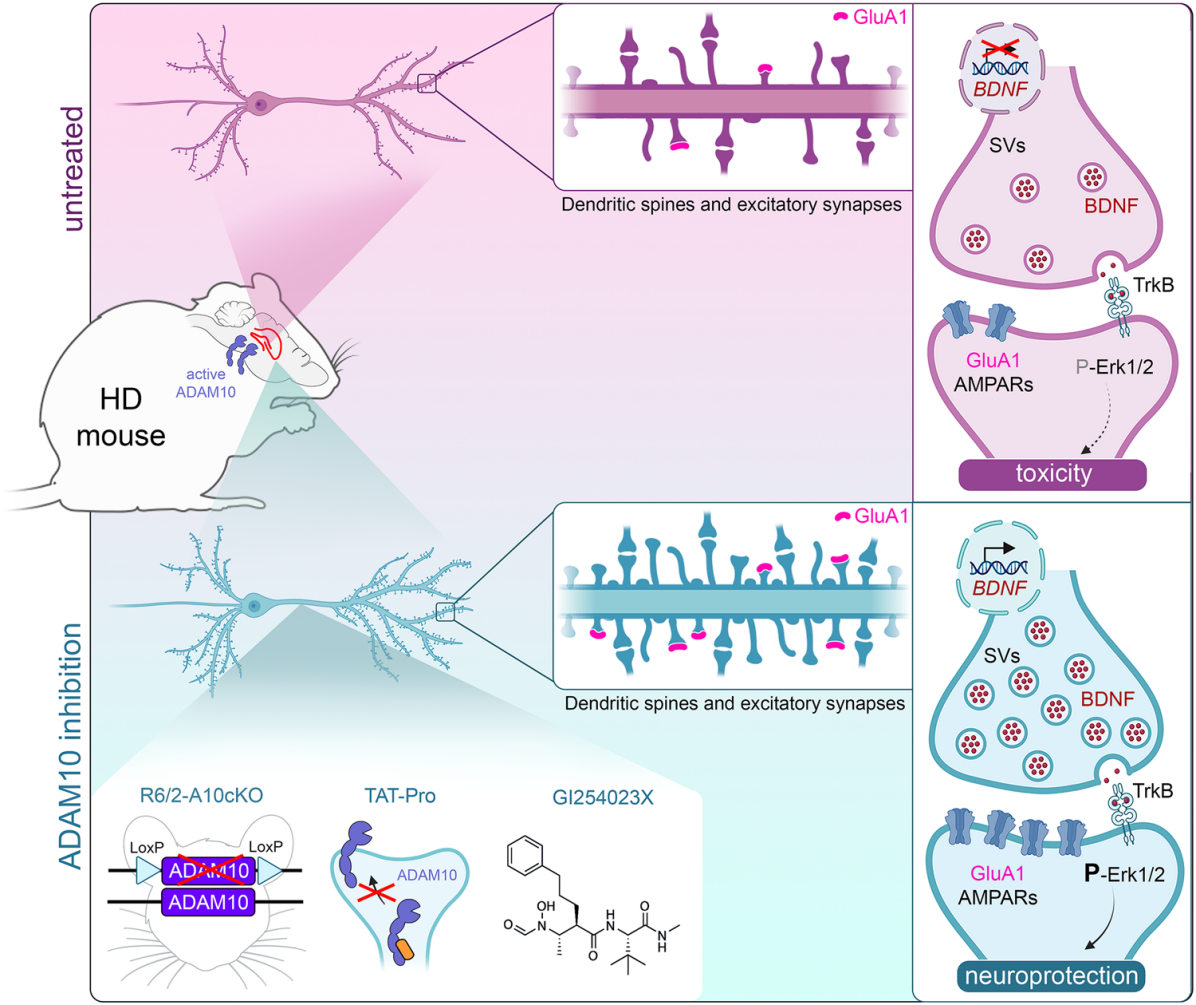
The ADAM10 and the BDNF/TrkB pathways at the HD hippocampal synapse. Defects in synaptic plasticity imply increased amounts of active ADAM10 in the HD hippocampus and downregulation of the BDNF/TrkB pathway. ADAM10 inhibition prevents the loss of long-lasting spines and enhances GluA1-AMPARs recruitment and LTP induction in mushroom spines, while also restoring BDNF and ERK signaling

Impaired cognitive function is a hallmark of HD and is often among the initial clinical symptoms. The cognitive challenges faced by patients, rather than the movement disorder, impose the greatest burden on HD families. Unfortunately, this aspect of HD pathology currently lacks a treatment. Progressive deficits in hippocampal-dependent cognition are observed in HD patients and have been correlated with estimated years to diagnosis in premanifest patients [12, 13, 61]. Our findings provide evidence that hyperactive ADAM10 strongly contributes to HD hippocampal synaptic defects (Fig. 7). We not only establish that active ADAM10 affects synaptic cell adhesion in the HD hippocampus, but we also reveal that the enzyme hyperactivity is linked to dysfunction of the BDNF/TrkB pathway. Collectively, these findings support ADAM10 inhibition as a valid therapeutic strategy to counter cognitive decline in HD.

### Supplementary Information

Below is the link to the electronic supplementary material.

Supplementary file1 (DOCX 1583 kb)

Supplementary file2 (DOCX 16 kb)

## Data Availability

The authors declare that all data supporting the findings of this study are available within the paper in the main text or the supplementary materials.

## References

[1] Bates GP, Dorsey R, Gusella JF, Hayden MR, Kay C, Leavitt BR, Nance M, Ross CA, Scahill RI, Wetzel R, Wild EJ, Tabrizi SJ (2015) Huntington disease. Nat Rev Dis Primers 1:15005. 10.1038/nrdp.2015.5 27188817 10.1038/nrdp.2015.5

[2] Paulsen JS, Langbehn DR, Stout JC, Aylward E, Ross CA, Nance M, Guttman M, Johnson S, MacDonald M, Beglinger LJ, Duff K, Kayson E, Biglan K, Shoulson I, Oakes D, Hayden M, Predict-HD Investigators and Coordinators of the Huntington Study Group (2008) Detection of Huntington’s disease decades before diagnosis: the Predict-HD study. J Neurol Neurosurg Psychiatry 79:874–880. 10.1136/jnnp.2007.128728 18096682 10.1136/jnnp.2007.128728 PMC2569211

[3] Duff K, Paulsen J, Mills J, Beglinger LJ, Moser DJ, Smith MM, Langbehn D, Stout J, Queller S, Harrington DL, PREDICT-HD Investigators and Coordinators of the Huntington Study Group (2010) Mild cognitive impairment in prediagnosed Huntington disease. Neurology 75:500–507. 10.1212/WNL.0b013e3181eccfa2 20610833 10.1212/WNL.0b013e3181eccfa2 PMC2918475

[4] Cepeda C, Wu N, André VM, Cummings DM, Levine MS (2007) The corticostriatal pathway in Huntington’s disease. Prog Neurobiol 81:253–721. 10.1016/j.pneurobio.2006.11.001 17169479 10.1016/j.pneurobio.2006.11.001 PMC1913635

[5] Veldman MB, Yang XW (2018) Molecular insights into cortico-striatal miscommunications in Huntington’s disease. Curr Opin Neurobiol 48:79–89. 10.1016/j.conb.2017.10.019 29125980 10.1016/j.conb.2017.10.019 PMC5825262

[6] Quirion JG, Parsons MP (2019) The onset and progression of hippocampal synaptic plasticity deficits in the Q175FDN mouse model of Huntington disease. Front Cell Neurosci 13:326. 10.3389/fncel.2019.00326 31379510 10.3389/fncel.2019.00326 PMC6650530

[7] Murphy KP, Carter RJ, Lione LA, Mangiarini L, Mahal A, Bates GP, Dunnett SB, Morton AJ (2000) Abnormal synaptic plasticity and impaired spatial cognition in mice transgenic for exon 1 of the human Huntington’s disease mutation. J Neurosci 20:5115–5123. 10.1523/JNEUROSCI.20-13-05115.2000 10864968 10.1523/JNEUROSCI.20-13-05115.2000 PMC6772265

[8] Anglada-Huguet M, Xifró X, Giralt A, Zamora-Moratalla A, Martín ED, Alberch J (2014) Prostaglandin E2 EP1 receptor antagonist improves motor deficits and rescues memory decline in R6/1 mouse model of Huntington’s disease. Mol Neurobiol 49:784–795. 10.1007/s12035-013-8556-x 24198227 10.1007/s12035-013-8556-x

[9] Giralt A, Brito V, Chevy Q, Simonnet C, Otsu Y, Cifuentes-Díaz C, de Pins B, Coura R, Alberch J, Ginés S, Poncer JC, Girault JA (2017) Pyk2 modulates hippocampal excitatory synapses and contributes to cognitive deficits in a Huntington’s disease model. Nat Commun 8:15592. 10.1038/ncomms15592 28555636 10.1038/ncomms15592 PMC5459995

[10] Zhang H, Zhang C, Vincent J, Zala D, Benstaali C, Sainlos M, Grillo-Bosch D, Daburon S, Coussen F, Cho Y, David DJ, Saudou F, Humeau Y, Choquet D (2018) Modulation of AMPA receptor surface diffusion restores hippocampal plasticity and memory in Huntington’s disease models. Nat Commun 9(1):4272. 10.1038/s41467-018-06675-3 30323233 10.1038/s41467-018-06675-3 PMC6189172

[11] Kraskovskaya NA, Erofeev AI, Grishina ED, Pushkareva SA, Gerasimov EI, Vlasova OL, Bezprozvanny IB (2021) Development of hippocampus-associated cognitive dysfunction in Huntington’s disease mouse model. J Evol Biochem Phys 57:1449–1460. 10.1134/S0022093021060211 10.1134/S0022093021060211

[12] Begeti F, Schwab LC, Mason SL, Barker RA (2016) Hippocampal dysfunction defines disease onset in Huntington’s disease. J Neurol Neurosurg Psychiatry 87:975–981. 10.1136/jnnp-2015-312413 26833174 10.1136/jnnp-2015-312413

[13] Harris KL, Armstrong M, Swain R, Erzinclioglu S, Das T, Burgess N, Barker RA, Mason SL (2019) Huntington’s disease patients display progressive deficits in hippocampal-dependent cognition during a task of spatial memory. Cortex 119:417–427. 10.1016/j.cortex.2019.07.014 31499434 10.1016/j.cortex.2019.07.014

[14] Zuccato C, Cattaneo E (2014) Huntington’s disease. Handb Exp Pharmacol 220:357–409. 10.1007/978-3-642-45106-5_14 24668480 10.1007/978-3-642-45106-5_14

[15] Lynch G, Kramar EA, Rex CS, Jia Y, Chappas D, Gall CM, Simmons DA (2007) Brain-derived neurotrophic factor restores synaptic plasticity in a knock-in mouse model of Huntington’s disease. J Neurosci 27:4424–4434. 10.1523/JNEUROSCI.5113-06.2007 17442827 10.1523/JNEUROSCI.5113-06.2007 PMC6672319

[16] Lo Sardo V, Zuccato C, Gaudenzi G, Vitali B, Ramos C, Tartari M, Myre MA, Walker JA, Pistocchi A, Conti L, Valenza M, Drung B, Schmidt B, Gusella J, Zeitlin S, Cotelli F, Cattaneo E (2012) An evolutionary recent neuroepithelial cell adhesion function of huntingtin implicates ADAM10-Ncadherin. Nat Neurosci 15:713–721. 10.1038/nn.3080 22466506 10.1038/nn.3080

[17] Saftig P, Lichtenthaler SF (2015) The alpha secretase ADAM10: a metalloprotease with multiple functions in the brain. Prog Neurobiol 135:1–20. 10.1016/j.pneurobio.2015.10.003 26522965 10.1016/j.pneurobio.2015.10.003

[18] Vezzoli E, Caron I, Talpo F, Besusso D, Conforti P, Battaglia E, Sogne E, Falqui A, Petricca L, Verani M, Martufi P, Caricasole A, Bresciani A, Cecchetti O, di Val R, Cervo P, Sancini G, Riess O, Nguyen H, Seipold L, Saftig P, Biella G, Cattaneo E, Zuccato C (2019) Inhibiting pathologically active ADAM10 rescues synaptic and cognitive decline in Huntington’s disease. J Clin Invest 129:2390–2403. 10.1172/JCI120616 31063986 10.1172/JCI120616 PMC6546448

[19] Cozzolino F, Vezzoli E, Cheroni C, Besusso D, Conforti P, Valenza M, Iacobucci I, Monaco V, Birolini G, Bombaci M, Falqui A, Saftig P, Rossi RL, Monti M, Cattaneo E, Zuccato C (2021) ADAM10 hyperactivation acts on piccolo to deplete synaptic vesicle stores in Huntington’s disease. Hum Mol Genet 30:1175–1187. 10.1093/hmg/ddab047 33601422 10.1093/hmg/ddab047

[20] Prox J, Bernreuther C, Altmeppen H, Grendel J, Glatzel M, D’Hooge R, Stroobants S, Ahmed T, Balschun D, Willem M, Lammich S, Isbrandt D, Schweizer M, Horré K, De Strooper B, Saftig P (2013) Postnatal disruption of the disintegrin/metalloproteinase ADAM10 in brain causes epileptic seizures, learning deficits, altered spine morphology, and defective synaptic functions. J Neurosci 32:12915–12928. 10.1523/JNEUROSCI.5910-12.2013 10.1523/JNEUROSCI.5910-12.2013 PMC6619719 23926248

[21] Vezzoli E, Calì C, De Roo M, Ponzoni L, Sogne E, Gagnon N, Francolini M, Braida D, Sala M, Muller D, Falqui A, Magistretti PJ (2020) Ultrastructural evidence for a role of astrocytes and glycogen-derived lactate in learning-dependent synaptic stabilization. Cereb Cortex 30:2114–2127. 10.1093/cercor/bhz226 31807747 10.1093/cercor/bhz226 PMC7174989

[22] Risher WC, Ustunkaya T, Singh Alvarado J, Eroglu C (2014) Rapid golgi analysis method for efficient and unbiased classification of dendritic spines. PLoS ONE 9(9):e107591. 10.1371/journal.pone.0107591 25208214 10.1371/journal.pone.0107591 PMC4160288

[23] Harris KM, Jensen FE, Tsao B (1992) Three-dimensional structure of dendritic spines and synapses in rat hippocampus (CA1) at postnatal day 15 and adult ages: implications for the maturation of synaptic physiology and long-term potentiation. J Neurosci 12:2685–2705. 10.1523/JNEUROSCI.12-07-02685.1992 1613552 10.1523/JNEUROSCI.12-07-02685.1992 PMC6575840

[24] Mangiarini L, Sathasivam K, Seller M, Cozens B, Harper A, Hetherington C, Lawton M, Trottier Y, Lehrach H, Davies SW, Bates GP (1996) Exon 1 of the HD gene with an expanded CAG repeat is sufficient to cause a progressive neurological phenotype in transgenic mice. Cell 87:493–506. 10.1016/s0092-8674(00)81369-0 8898202 10.1016/s0092-8674(00)81369-0

[25] Birolini G, Valenza M, Di Paolo E, Vezzoli E, Talpo F, Maniezzi C, Caccia C, Leoni V, Taroni F, Bocchi VD, Conforti P, Sogne E, Petricca L, Cariulo C, Verani M, Caricasole A, Falqui A, Biella G, Cattaneo E (2020) Striatal infusion of cholesterol promotes dose-dependent behavioral benefits and exerts disease- modifying effects in Huntington’s disease mice. EMBO Mol Med 12(10):e12519. 10.15252/emmm.202012519 32959531 10.15252/emmm.202012519 PMC7539329

[26] Etxeberria-Rekalde E, Alzola-Aldamizetxebarria S, Flunkert S, Hable I, Daurer M, Neddens J, Hutter-Paier B (2021) Quantification of Huntington’s disease related markers in the R6/2 mouse model. Front Mol Neurosci 13:617229. 10.3389/fnmol.2020.617229 33505246 10.3389/fnmol.2020.617229 PMC7831778

[27] Menalled LB, Kudwa AE, Miller S, Fitzpatrick J, Watson-Johnson J, Keating N, Ruiz M, Mushlin R, Alosio W, McConnell K, Connor D, Murphy C, Oakeshott S, Kwan M, Beltran J, Ghavami A, Brunner D, Park LC, Ramboz S, Howland D (2012) Comprehensive behavioral and molecular characterization of a new knock-in mouse model of Huntington’s disease: zQ175. PLoS ONE 7(12):e49838. 10.1371/journal.pone.0049838 23284626 10.1371/journal.pone.0049838 PMC3527464

[28] Asada-Utsugi M, Uemura K, Kubota M, Noda Y, Tashiro Y, Uemura TM, Yamakado H, Urushitani M, Takahashi R, Hattori S, Miyakawa T, Ageta-Ishihara N, Kobayashi K, Kinoshita M, Kinoshita A (2021) Mice with cleavage-resistant N-cadherin exhibit synapse anomaly in the hippocampus and outperformance in spatial learning tasks. Mol Brain 14(1):23. 10.1186/s13041-021-00738-1 33494786 10.1186/s13041-021-00738-1 PMC7831172

[29] Reiss K, Maretzky T, Ludwig A, Tousseyn T, de Strooper B, Hartmann D, Saftig P (2005) ADAM10 cleavage of N-cadherin and regulation of cell-cell adhesion and beta- catenin nuclear signalling. EMBO J 24:742–752. 10.1038/sj.emboj.7600548 15692570 10.1038/sj.emboj.7600548 PMC549617

[30] Bulley SJ, Drew CJ, Morton AJ (2012) Direct visualisation of abnormal dendritic spine morphology in the hippocampus of the R6/2 transgenic mouse model of huntington’s disease. J Huntingtons Dis 1:267–273. 10.3233/JHD-120024 25063335 10.3233/JHD-120024

[31] Chanaday NL, Cousin MA, Milosevic I, Watanabe S, Morgan JR (2019) The synaptic vesicle cycle revisited: new insights into the modes and mechanisms. J Neurosci 39:8209–8216. 10.1523/JNEUROSCI.1158-19.2019 31619489 10.1523/JNEUROSCI.1158-19.2019 PMC6794917

[32] Droogers WJ, MacGillavry HD (2023) Plasticity of postsynaptic nanostructure. Mol Cell Neurosci 124:103819. 10.1016/j.mcn.2023.103819 36720293 10.1016/j.mcn.2023.103819

[33] Zuccato C, Ciammola A, Rigamonti D, Leavitt BR, Goffredo D, Conti L, MacDonald ME, Friedlander RM, Silani V, Hayden MR, Timmusk T, Sipione S, Cattaneo E (2001) Loss of huntingtin-mediated BDNF gene transcription in Huntington’s disease. Science 293:493–498. 10.1126/science.1059581 11408619 10.1126/science.1059581

[34] Zuccato C, Tartari M, Crotti A, Goffredo D, Valenza M, Conti L, Cataudella T, Leavitt BR, Hayden MR, Timmusk T, Rigamonti D, Cattaneo E (2003) Huntingtin interacts with REST/NRSF to modulate the transcription of NRSE-controlled neuronal genes. Nat Genet 35:76–83. 10.1038/ng1219 12881722 10.1038/ng1219

[35] Gauthier LR, Charrin BC, Borrell-Pagès M, Dompierre JP, Rangone H, Cordelières FP, De Mey J, MacDonald ME, Lessmann V, Humbert S, Saudou F (2004) Huntingtin controls neurotrophic support and survival of neurons by enhancing BDNF vesicular transport along microtubules. Cell 118:127–138. 10.1016/j.cell.2004.06.018 15242649 10.1016/j.cell.2004.06.018

[36] Zuccato C, Belyaev N, Conforti P, Ooi L, Tartari M, Papadimou E, MacDonald M, Fossale E, Zeitlin S, Buckley N, Cattaneo E (2007) Widespread disruption of repressor element-1 silencing transcription factor/neuron-restrictive silencer factor occupancy at its target genes in Huntington’s disease. J Neurosci 27:6972–6983. 10.1523/JNEUROSCI.4278-06.2007 17596446 10.1523/JNEUROSCI.4278-06.2007 PMC6672230

[37] Leal G, Afonso PM, Salazar IL, Duarte CB (2015) Regulation of hippocampal synaptic plasticity by BDNF. Brain Res 1621:82–101. 10.1016/j.brainres.2014.10.019 25451089 10.1016/j.brainres.2014.10.019

[38] Simmons DA, Belichenko NP, Yang T, Condon C, Monbureau M, Shamloo M, Jing D, Massa SM, Longo FM (2013) A small molecule TrkB ligand reduces motor impairment and neuropathology in R6/2 and BACHD mouse models of Huntington’s disease. J Neurosci 33:18712–18727. 10.1523/JNEUROSCI.1310-13.2013 24285878 10.1523/JNEUROSCI.1310-13.2013 PMC3841443

[39] Nguyen KQ, Rymar VV, Sadikot AF (2016) Impaired TrkB signaling underlies reduced BDNF-mediated trophic support of striatal neurons in the R6/2 mouse model of Huntington’s disease. Front Cell Neurosci 10:37. 10.3389/fncel.2016.00037 27013968 10.3389/fncel.2016.00037 PMC4783409

[40] Aid T, Kazantseva A, Piirsoo M, Palm K, Timmusk T (2007) Mouse and rat BDNF gene structure and expression revisited. J Neurosci Res 85:525–535. 10.1002/jnr.21139 17149751 10.1002/jnr.21139 PMC1878509

[41] Zuccato C, Liber D, Ramos C, Tarditi A, Rigamonti D, Tartari M, Valenza M, Cattaneo E (2005) Progressive loss of BDNF in a mouse model of Huntington’s disease and rescue by BDNF delivery. Pharmacol Res 52:133–139. 10.1016/j.phrs.2005.01.001 15967378 10.1016/j.phrs.2005.01.001

[42] Benn CL, Fox H, Bates GP (2008) Optimisation of region-specific reference gene selection and relative gene expression analysis methods for pre-clinical trials of Huntington’s disease. Mol Neurodegener 3:17. 10.1186/1750-1326-3-17 18954449 10.1186/1750-1326-3-17 PMC2584034

[43] Alonso M, Medina JH, Pozzo-Miller L (2008) ERK1/2 activation is necessary for BDNF to increase dendritic spine density in hippocampal CA1 pyramidal neurons. Learn Mem 11:172–178. 10.1101/lm.67804 10.1101/lm.67804 PMC379687 15054132

[44] Marcello E, Gardoni F, Mauceri D, Romorini S, Jeromin A, Epis R, Borroni B, Cattabeni F, Sala C, Padovani A, Di Luca M (2007) Synapse-associated protein-97 mediates alpha-secretase ADAM10 trafficking and promotes its activity. J Neurosci 27:1682–1691. 10.1523/JNEUROSCI.3439-06.2007 17301176 10.1523/JNEUROSCI.3439-06.2007 PMC6673742

[45] Ludwig A, Hundhausen C, Lambert MH, Broadway N, Andrews RC, Bickett DM, Leesnitzer MA, Becherer JD (2005) Metalloproteinase inhibitors for the disintegrin- like metalloproteinases ADAM10 and ADAM17 that differentially block constitutive and phorbol ester-inducible shedding of cell surface molecules. Comb Chem High Throughput Screen 8:161–171. 10.2174/1386207053258488 15777180 10.2174/1386207053258488

[46] Hoettecke N, Ludwig A, Foro S, Schmidt B (2010) Improved synthesis of ADAM10 inhibitor GI254023X. Neurodegener Dis 7:232–238. 10.1159/000267865 20197648 10.1159/000267865

[47] Cazorla M, Prémont J, Mann A, Girard N, Kellendonk C, Rognan D (2011) Identification of a low-molecular weight TrkB antagonist with anxiolytic and antidepressant activity in mice. J Clin Invest 121:1846–1857. 10.1172/JCI43992 21505263 10.1172/JCI43992 PMC3083767

[48] Runge K, Cardoso C, de Chevigny A (2020) Dendritic spine plasticity: function and mechanisms. Front Synaptic Neurosci 12:36. 10.3389/fnsyn.2020.00036 32982715 10.3389/fnsyn.2020.00036 PMC7484486

[49] Verstraelen P, Garcia-Diaz Barriga G, Verschuuren M, Asselbergh B, Nuydens R, Larsen PH, Timmermans JP, De Vos WH (2020) Systematic quantification of synapses in primary neuronal culture. iScience 23(9):101542. 10.1016/j.isci.2020.101542 33083769 10.1016/j.isci.2020.101542 PMC7516133

[50] Fortin DA, Davare MA, Srivastava T, Brady JD, Nygaard S, Derkach VA, Soderling TR (2010) Long-term potentiation-dependent spine enlargement requires synaptic Ca2+-permeable AMPA receptors recruited by CaM-kinase I. J Neurosci 30:11565–11575. 10.1523/JNEUROSCI.1746-10.2010 20810878 10.1523/JNEUROSCI.1746-10.2010 PMC2943838

[51] Ovtscharoff W Jr, Segal M, Goldin M, Helmeke C, Kreher U, Greenberger V, Herzog A, Michaelis B, Braun K (2008) Electron microscopic 3D-reconstruction of dendritic spines in cultured hippocampal neurons undergoing synaptic plasticity. Dev Neurobiol 68:870–876. 10.1002/dneu.20627 18327766 10.1002/dneu.20627

[52] Diering GH, Huganir RL (2018) The AMPA receptor code of synaptic plasticity. Neuron 100:314–329. 10.1016/j.neuron.2018.10.018 30359599 10.1016/j.neuron.2018.10.018 PMC6214363

[53] Choquet D, Hosy E (2020) AMPA receptor nanoscale dynamic organization and synaptic plasticities. Curr Opin Neurobiol 63:137–145. 10.1016/j.conb.2020.04.003 32416471 10.1016/j.conb.2020.04.003

[54] Lynch MA (2004) Long-term potentiation and memory. Physiol Rev 84:87–136. 10.1152/physrev.00014.2003 14715912 10.1152/physrev.00014.2003

[55] Nakata H, Nakamura S (2007) Brain-derived neurotrophic factor regulates AMPA receptor trafficking to post-synaptic densities via IP3R and TRPC calcium signaling. FEBS Lett 581:2047–2054. 10.1016/j.febslet.2007.04.041 17482902 10.1016/j.febslet.2007.04.041

[56] Rosenbaum D, Saftig P (2023) New insights into the function and pathophysiology of the ectodomain sheddase a disintegrin and metalloproteinase 10 (ADAM10). FEBS J 291:2733–2766. 10.1111/febs.16870 37218105 10.1111/febs.16870

[57] West AE, Pruunsild P, Timmusk T (2014) Neurotrophins: transcription and translation. Handb Exp Pharmacol 220:67–100. 10.1007/978-3-642-45106-5_4 24668470 10.1007/978-3-642-45106-5_4

[58] Nishihara S, Tsuda L, Ogura T (2003) The canonical Wnt pathway directly regulates NRSF/REST expression in chick spinal cord. Biochem Biophys Res Commun 311:55–63. 10.1016/j.bbrc.2003.09.158 14575694 10.1016/j.bbrc.2003.09.158

[59] de Souza JM, Abd-Elrahman KS, Ribeiro FM, Ferguson SSG (2020) mGluR5 regulates REST/NRSF signaling through N-cadherin/β-catenin complex in Huntington’s disease. Mol Brain 13(1):118. 10.1186/s13041-020-00657-7 32859226 10.1186/s13041-020-00657-7 PMC7456045

[60] You H, Lu B (2023) Diverse functions of multiple Bdnf transcripts driven by distinct Bdnf promoters. Biomolecules 13(4):655. 10.3390/biom13040655 37189402 10.3390/biom13040655 PMC10135494

[61] Wilkes FA, Jakabek D, Walterfang M, Velakoulis D, Poudel GR, Stout JC, Chua P, Egan GF, Looi JCL, Georgiou-Karistianis N (2023) Hippocampal morphology in Huntington’s disease, implications for plasticity and pathogenesis: The IMAGE-HD study. Psychiatry Res Neuroimaging 335:111694. 10.1016/j.pscychresns.2023.111694 37598529 10.1016/j.pscychresns.2023.111694

