# Supplementary material for: Neuroprotection by ADAM10 inhibition requires TrkB signaling in the Huntington’s disease hippocampus
Source: Cell Mol Life Sci. 2024 Aug 7;81(1):333. doi: 10.1007/s00018-024-05382-1 (PMC11335257; doi:10.1007/s00018-024-05382-1)
Supplement: Supplementary file 1 — Supplementary file1 (DOCX 1583 kb) [file 18_2024_5382_MOESM1_ESM.docx]

| Multiple comparisons | Summary | Adjusted P Value |
| --- | --- | --- |
| Basal WT vs. Basal R6/2 | *** | 0,0002 |
| Basal WT vs. Basal R6/2+GI+ANA12 | * | 0,0253 |
| Basal WT vs. cLTP R6/2 | **** | <0,0001 |
| Basal WT vs. cLTP R6/2+GI+ANA12 | * | 0,0228 |
| Basal R6/2 vs. Basal R6/2+GI | * | 0,0153 |
| Basal R6/2 vs. cLTP WT | *** | 0,0008 |
| Basal R6/2+GI vs. cLTP R6/2 | **** | <0,0001 |
| cLTP WT vs. cLTP R6/2 | **** | <0,0001 |
| cLTP R6/2 vs. cLTP R6/2+GI | ** | 0,0021 |

**Supplementary Table 1.** Summary of statistical analyses related to Fig. 6F. Two-way ANOVA with Tukey’s post-hoc test. GraphPad Prism Version 9.4.0 (453).

| Multiple comparisons | Summary | Adjusted P Value |
| --- | --- | --- |
| Basal WT vs. cLTP WT | *** | 0,0010 |
| Basal WT vs. cLTP R6/2 | ** | 0,0077 |
| Basal WT vs. cLTP R6/2+GI | **** | <0,0001 |
| Basal WT+ANA12 vs. cLTP R6/2 | ** | 0,0022 |
| Basal WT+ANA12 vs. cLTP R62+GI | * | 0,0271 |
| Basal R6/2 vs. cLTP WT | *** | 0,0009 |
| Basal R6/2 vs. cLTP R6/2+GI | **** | <0,0001 |
| Basal R6/2+ANA12 vs. cLTP WT | **** | <0,0001 |
| Basal R6/2+ANA12 vs. cLTP R6/2+GI | **** | <0,0001 |
| Basal R6/2+GI vs. cLTP R6/2 | * | 0,0448 |
| Basal R6/2+GI vs. cLTP R6/2+GI | * | 0,0171 |
| Basal R6/2+GI+ANA12 vs. cLTP WT | * | 0,0382 |
| Basal R6/2+GI+ANA12 vs. cLTP R6/2+GI | ** | 0,0023 |
| cLTP WT vs. cLTP WT+ANA12 | ** | 0,0014 |
| cLTP WT vs. cLTP R6/2 | **** | <0,0001 |
| cLTP WT vs. cLTP R6/2+ANA12 | **** | <0,0001 |
| cLTP WT+ANA12 vs. cLTP R6/2+GI | **** | <0,0001 |
| cLTP R6/2 vs. cLTP R6/2+GI | **** | <0,0001 |
| cLTP R6/2 vs. cLTP R6/2+GI+ANA12 | ** | 0,0050 |
| cLTP R6/2+ANA12 vs. cLTP R6/2+GI | **** | <0,0001 |
| cLTP R62+GI vs. cLTP R6/2+GI+ANA12 | * | 0,0438 |

**Supplementary Table 2.** Summary of statistical analyses related to Fig. 6G. Two-way ANOVA with Tukey’s post-hoc test. GraphPad Prism Version 9.4.0 (453).
