# Supplementary material for: Neuroprotection by ADAM10 inhibition requires TrkB signaling in the Huntington’s disease hippocampus
Source: Cell Mol Life Sci. 2024 Aug 7;81(1):333. doi: 10.1007/s00018-024-05382-1 (PMC11335257; doi:10.1007/s00018-024-05382-1)
Supplement: Supplementary file 2 — Supplementary file2 (DOCX 16 kb) [file 18_2024_5382_MOESM2_ESM.docx]

WT


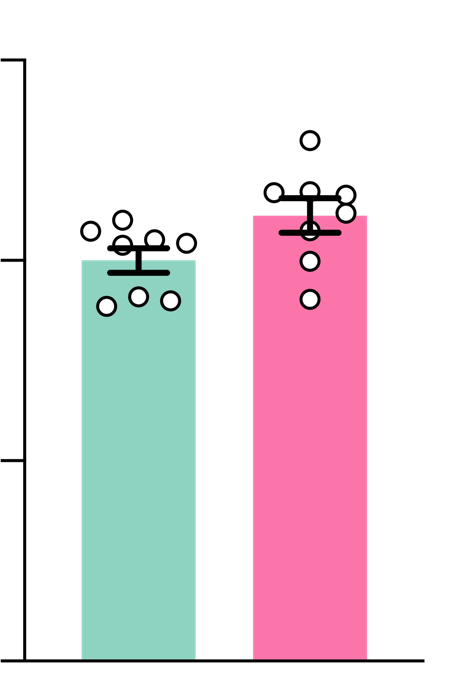


150

R6/2

50

0

100

## Supplementary Figure 1. ADAM10 mRNA level in the hippocampus of WT and R6/2 mice

ADAM10/β-actin mRNA

% of WT

The ADAM10 mRNA level was determined in the hippocampus of WT and R6/2 mice at 10-12 weeks of age by RT-qPCR and normalized to the level of β-Actin mRNA. Data are represented as the mean ± SEM and were analyzed by unpaired t test. n=8 mice/genotype were used.

# A

WT R6/2

B

200


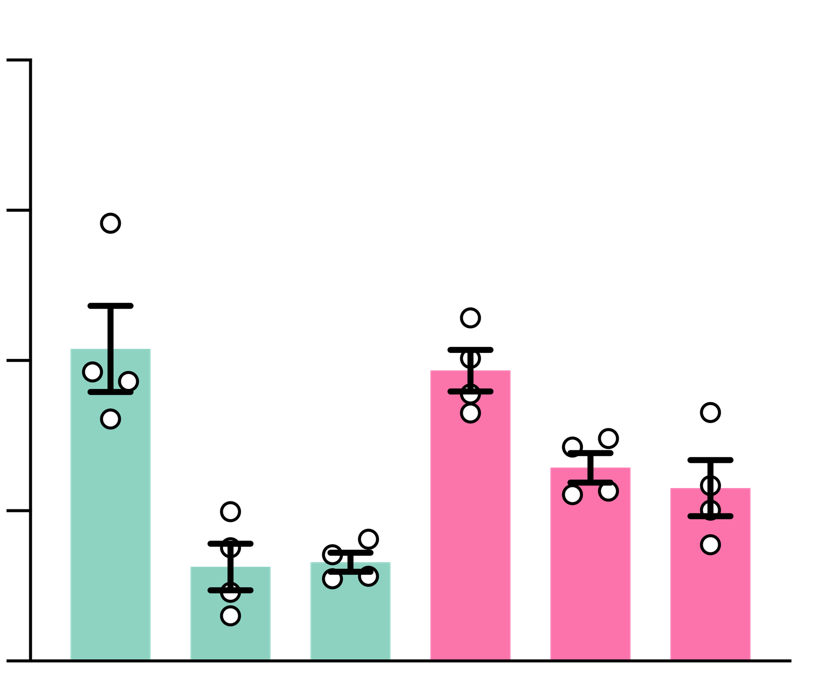


~~*~~

~~*~~

m-ADAM10/β-III-Tubulin

% of DG-WT

150

WT R6/2

kDa DG CA1 CA3 DG CA1 CA3 75 -


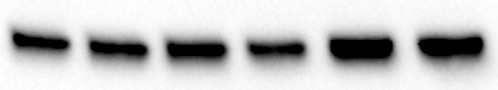

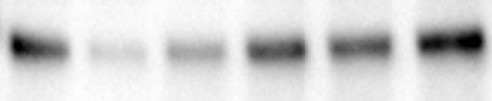


m-ADAM10

100

50 -

β-III-Tubulin

50

0

DG CA1 CA3 DG CA1 CA3

## Supplementary Figure 2. The mature active form of ADAM10 is increased in HD CA1 and CA3

(**A**) Representative Western Blot of m-ADAM10 in the DG, CA1 and CA3 hippocampal regions of WT and R6/2 mice at 12 weeks of age. β-III-Tubulin, loading control. (**B**) Quantification of data shown in A. n=4 mice/genotype. Data are represented as mean ± SEM. *P<0.05 unpaired t test.

# A

WT R6/2 R6/2-A10cKO

B

400
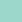
 WT

m-ADAM10/β-3-III-Tubulin

% of WT


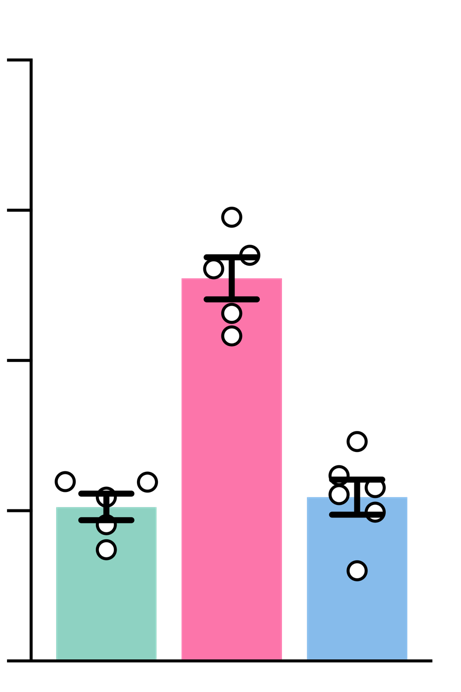


~~****~~   ~~****~~

kDa 75 -

50 -

#1 #2 #1 #2

#1 #2 #3

m-ADAM10


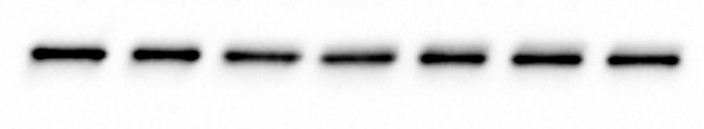

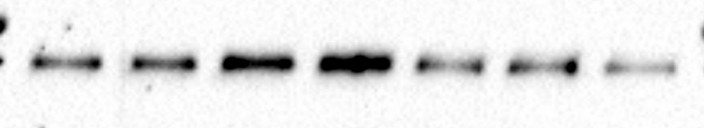


β-III-Tubulin

300

200

100

R6/2

R6/2-A10cKO

0

## Supplementary Figure 3. Synaptic level of the active form of ADAM10 in the hippocampus of R6/2-A10cKO mice

CaMKIIα-Cre:Adam10Flox/+(A10cKO) mice were crossed with R6/2 mice to obtain R6/2:CaMKIIα-Cre:Adam10Flox/+ mice (R6/2-A10cKO). (**A**) Representative Western blot performed with an antibody able to detect the mature active form of ADAM10 (m-ADAM10) in the hippocampus of WT, R6/2, and R6/2-A10cKO at 13 weeks of age. β-III-Tubulin, loading control. (**B**) Quantification of data in A. n=5-6 mice/genotype. Data are represented as mean ± SEM. ****P < 0.0001, One-way ANOVA with Tukey’s post hoc test.


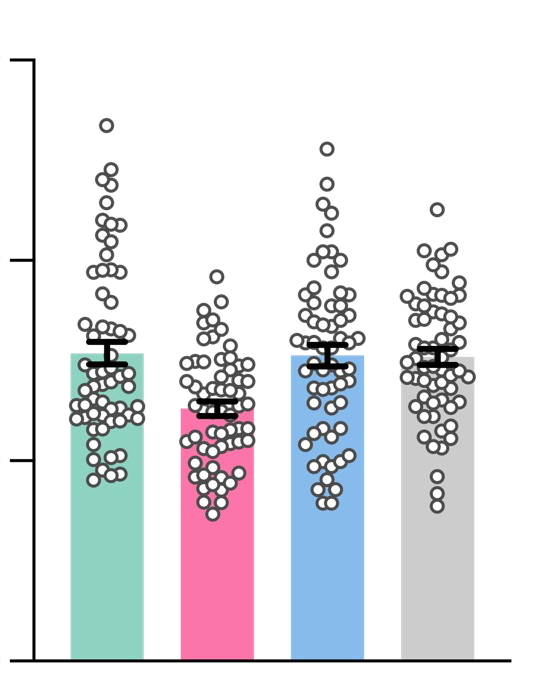


~~***~~

~~***~~

~~***~~

300

200

PSD length

WT R6/2

R6/2-A10cKO A10cKO

100

nm

0

## Supplementary Figure 4. Normalization of the active ADAM10 level prevents post-synaptic defects in R6/2 hippocampal neurons

Morphometric analysis of post-synaptic density (PSD) length in hippocampal CA1 pyramidal neurons of WT, R6/2, R6/2- A10cKO, and A10cKO mice. 60 PSDs were analyzed in n=3 mice/genotype at 13 weeks of age. Data are presented as mean ± SEM. ***P<0.001, Kruskal-Wallis followed by Dunn’s multiple comparison test.

# A

TAT-Ala-ADAM10709-729 TAT-Pro-ADAM10709-729


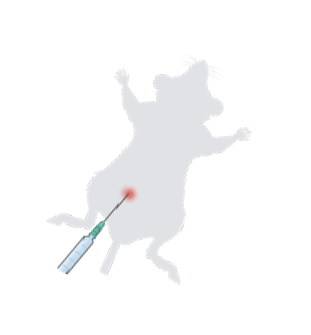

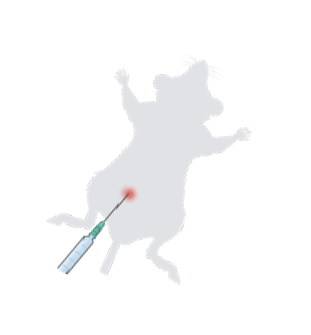

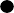

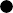

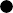


t=0 t=24h

# B

t=48h sacrifice

KDa

#1 #2 #3 #1 #2 #3 #4 #1 #2 #3

50 -


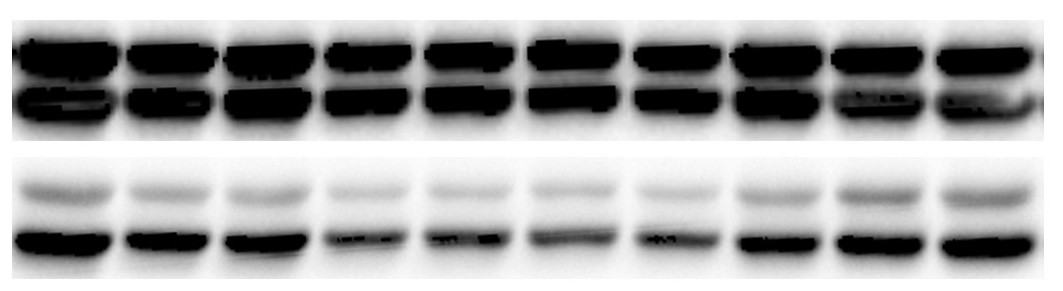

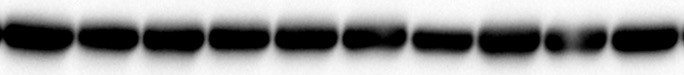


50 -

50 -

WT-TAT-Ala

R6/2-TAT-Ala R6/2-TAT-Pro

ERK1-T ERK2-T

ERK1-P ERK2-P

𝜶-Tubulin

# C D


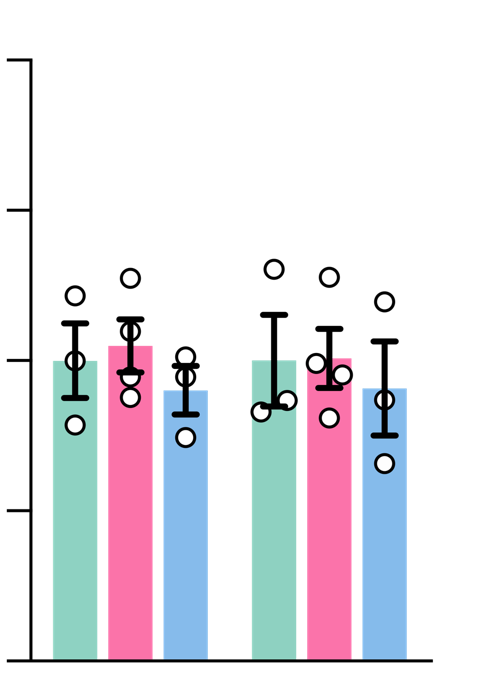


WT-TAT-Ala

R6/2-TAT-Ala

200

R6/2-TAT-Pro

150

100

50

0

200


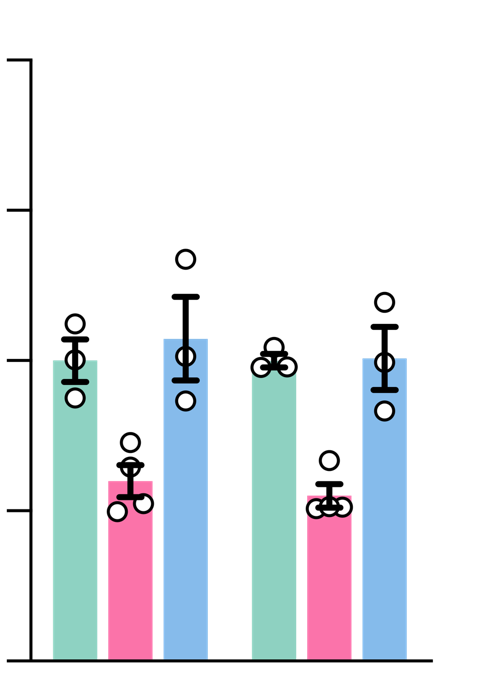


~~*~~

~~*~~

~~**~~

~~**~~

150

ERK-T/α-Tubulin

% of WT

100

ERK-P/ERK-T

% of WT

50

0

ERK1-P ERK2-P ERK1-T ERK2-T

## Supplementary Figure 5. Acute exposure to TAT-Pro increases P-ERK1 and P-ERK2 in R6/2 mice

(**A**) TAT-Pro-ADAM10709–729 (TAT-Pro) interferes with ADAM10/SAP97 interaction and impairs ADAM10 synaptic trafficking. Specifically, TAT-Pro contains the poly-proline stretch of ADAM10 required for binding to SAP97 and, by sequestering SAP97, it blocks SAP97-mediated ADAM10 trafficking to the synapse and its activity. As control, the analogous inactive peptide TAT-Ala-ADAM10709–729, in which all proline residues were substituted by alanines, was used. 12-week-old WT and R6/2 mice received 2 i.p. injections of TAT-Pro or TAT-Ala at 2 nmol/g 24 hours apart. P-ERK1/2 was monitored 24 hours after the second i.p. injection. (**B**) Representative Western blot of phosphorylated and total ERK1/2 in the hippocampus of WT-TAT-Ala, R6/2-TAT-Ala and R6/2-TAT-Pro. α-Tubulin, loading control. **(C,D**) Quantification of data shown in B. WT-TAT-Ala, n=3; R6/2-TAT-Ala, n=4; R6/2-TAT-Pro, n=3. Data are represented as mean ± SEM. *P < 0.05; **P < 0.01, Ordinary one-way ANOVA with Tukey’s post hoc test.

WT R6/2

R6/2 + GI

R6/2 + GI + ANA12

# A B C

membrane capacitance sEPSCs rise time sEPSCs decay time


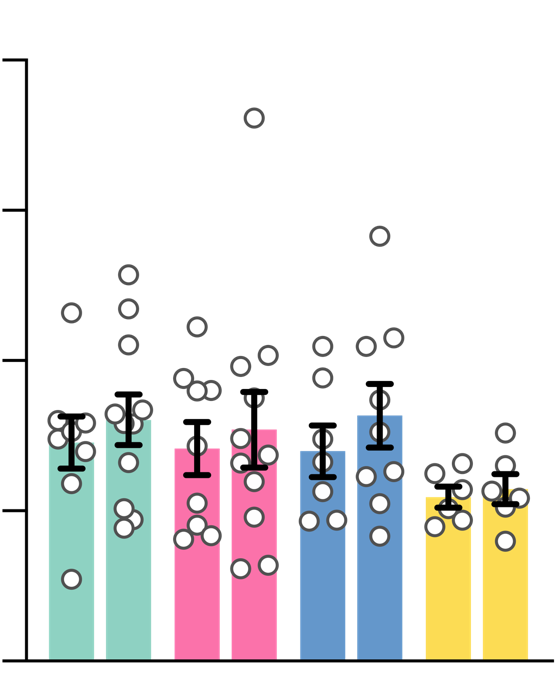

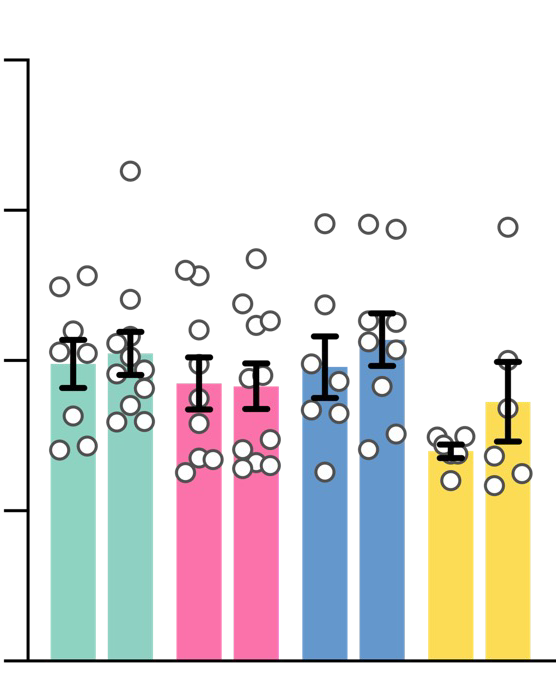

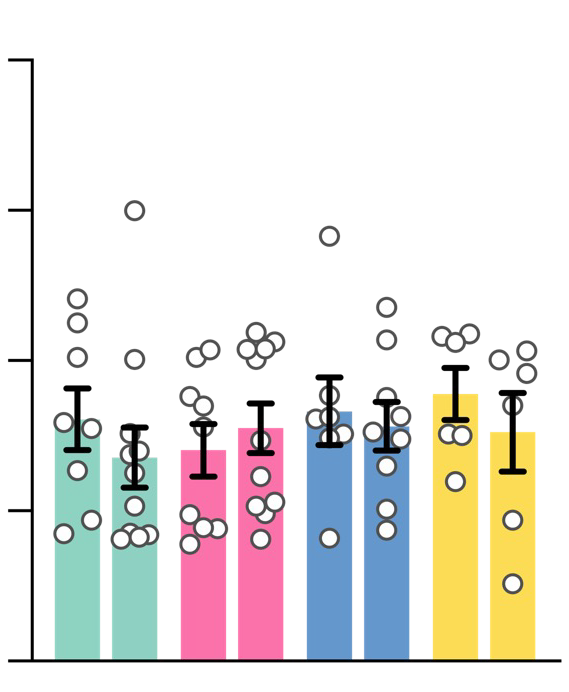


200 8 40

150 6 30

100 4 20

pF

ms

ms

50 2 10

0

cLTP - +

- + - + - +

0

cLTP - +

- + - + - +

0

cLTP - +

- + - + - +

## Supplementary Figure 6. Membrane capacitance, sEPSCs rise and decay times in WT and R6/2 hippocampal neurons before and after cLTP induction

The ADAM10 inhibitor GI254023X (GI, 1 µM) was administered from DIV6 until DIV14. The TrkB antagonist ANA12 (10 µM) was administered at DIV12 until DIV14. Chemical LTP was induced at DIV14 with 0.2 mM glycine for 15 minutes. (**A**) Membrane capacitance. (**B**) sEPSCs rise time. (**C**) sEPSCs decay time. Data are expressed as mean ± SEM and were analyzed by Two-way ANOVA followed by Bonferroni’s post hoc test.
